# Towards a unified medical microbiome ecology of the OMU for metagenomes and the OTU for microbes

**DOI:** 10.1186/s12859-023-05591-8

**Published:** 2024-03-29

**Authors:** Zhanshan (Sam) Ma

**Affiliations:** 1 Computational Biology and Medical Ecology Lab, State Key Lab of Genetic Resources and Evolution, Center for Excellence in Animal Evolution and Genetics, Kunming Institute of Zoology, Chinese Academy of Sciences, Kunming, China; 2 Microbiome Medicine and Advanced AI Lab, Cambridge, MA 02138 USA; 3 Faculty of Arts and Science, Harvard University, Cambridge, MA 02138 USA

**Keywords:** Operational metagenomic unit (OMU), Operational taxonomic unit (OTU), Metagenome ecology, Unified ecology of microbiomes and macrobiomes, Sloan near neutral model, Core/periphery network, High-salience skeleton network, Unified ecology of metagenomes and organisms (species), Medical ecology

## Abstract

**Background:**

Metagenomic sequencing technologies offered unprecedented opportunities and also challenges to microbiology and microbial ecology particularly. The technology has revolutionized the studies of microbes and enabled the high-profile human microbiome and earth microbiome projects. The terminology-change from microbes to microbiomes signals that our capability to count and classify microbes (*microbiomes*) has achieved the same or similar level as we can for the biomes (*macrobiomes*) of plants and animals (macrobes). While the traditional investigations of macrobiomes have usually been conducted through naturalists’ (Linnaeus & Darwin) naked eyes, and aerial and satellite images (remote-sensing), the large-scale investigations of microbiomes have been made possible by DNA-sequencing-based metagenomic technologies. Two major types of metagenomic sequencing technologies—amplicon sequencing and whole-genome (shotgun sequencing)—respectively generate two contrastingly different categories of metagenomic reads (data)—OTU (operational taxonomic unit) tables representing microorganisms and OMU (operational metagenomic unit), a new term coined in this article to represent various cluster units of metagenomic genes.

**Results:**

The ecological science of microbiomes based on the OTU representing microbes has been unified with the classic ecology of macrobes (macrobiomes), but the unification based on OMU representing metagenomes has been rather limited. In a previous series of studies, we have demonstrated the applications of several classic ecological theories (diversity, composition, heterogeneity, and biogeography) to the studies of metagenomes. Here I push the envelope for the unification of OTU and OMU again by demonstrating the applications of metacommunity assembly and ecological networks to the metagenomes of human gut microbiomes. Specifically, the neutral theory of biodiversity (Sloan’s near neutral model), Ning et al.stochasticity framework, core-periphery network, high-salience skeleton network, special trio-motif, and positive-to-negative ratio are applied to analyze the OMU tables from whole-genome sequencing technologies, and demonstrated with seven human gut metagenome datasets from the human microbiome project.

**Conclusions:**

All of the ecological theories demonstrated previously and in this article, including diversity, composition, heterogeneity, stochasticity, and complex network analyses, are equally applicable to OMU metagenomic analyses, just as to OTU analyses. Consequently, I strongly advocate the unification of OTU/OMU (microbiomes) with classic ecology of plants and animals (macrobiomes) in the context of medical ecology.

**Supplementary Information:**

The online version contains supplementary material available at 10.1186/s12859-023-05591-8.

## Background

The repertoire for post-sequencing-reads pipeline analyses for metagenome data from whole-genome sequencing technology (also known as shotgun sequencing) is much smaller than those for the OTU (operational taxonomic unit) data from amplicon sequencing (e.g., 16s-rRNA) for two main reasons. The first is the uncertainty around the applicability of ecological theories given that metagenomes are assemblages of genes, rather than of organisms. Second, orders of magnitude more MGs (metagenomic genes) than the numbers of microbial species make the analyses, especially ecological network analysis, extremely challenging computationally. I demonstrate the applicability of a set of ecological/network approaches, including Sloan near-neutral model, core/periphery network, high-salience skeleton network, trio-motif and PN ratio for assessing and interpreting the relative significance of the four processes (drift, selection, mutation, dispersal) in shaping the assemblage patterns of metagenomes (similar to metacommunity of organisms or species). In our opinion, the time has come for accelerated efforts towards a unified medical ecology of microbiomes/macrobiomes and organisms/metagenomes by filling historical gaps among ecology, genetics, and biomedicines.

Neutral theory originated in studies on gene mutation in molecular evolution [1, 2], and it was later extended to the field of community ecology and biogeography [3]. The neutral theory of evolution maintains that most variations at the molecular level do not have significant impacts on evolutionary fitness, and consequently the fate of genetic variations are mostly interpreted by stochastic drifts [4]. In the 1990s, Stephen Hubbell introduced the ecological neutral theory for interpreting biodiversity and biogeography by assuming that organisms from different species are *equivalent* with each other, and their differences in demography (birth, death, and migration) are stochastic drifts [3]. However, a perfect neutral theory would contradict the principles of classic niche selection, which suggests that organisms of different species usually only live and prosper in their own niches. In other words, it is the habitat (niche) properties that determine (select) the composition of ecological community, rather than stochastic drifts as neutral theory predicts. In both fields of molecular evolution and biodiversity, neutral models have successfully acted as null models for assessing and interpreting the relative importance of stochastic drifts versus deterministic selections. Furthermore, in both fields, it is postulated that there are four classes of process including *drift*, *selection*, *speciation* (*mutation*), and *dispersal* (gene flow), which constitute the critical processes that shape community dynamics and biogeography (synthesis of community ecology) or patterns of genetic variation (synthesis of population genetics) [5–8]. Neutral theory models explicitly consider three of the four above-mentioned processes (excluding selection), and therefore offer potential tools for examining the significance of those processes, particularly if complemented with other tools that can effectively measure the effects of selection forces.

Inspired by the above-mentioned success of neutral theory in community ecology and population genetics, the present study is aimed to explore its extensions to the domain of ‘assemblage of metagenomes.’ An assemblage of metagenomes is similar to an assemblage of communities (i.e., metacommunity) in community ecology [9–11]. For example, within a family (or cohort), each family (cohort) member is inhabited by his or her microbiome, and the total microbial genes carried by the microbiome can be defined as the individual’s metagenome. For the family (cohort), on the one hand, the assemblage (collection) of all microbial communities is known as a metacommunity in the vocabulary of community ecology; on the other hand, the ‘assemblage of metagenomes’ can be considered as the counterpart of ecological metacommunity [9–11]. An important assumption in either metacommunity or metagenome assemblage is the exchange (dispersal or migration) of either microbes or MGs among individuals (family or cohort members). In the above description of family (cohort) setting, the ‘scale’ of local community (metagenome) is individual, and consequently the metacommunity (metagenome assemblage) is defined for a family (cohort) or any population entity with individual or gene exchanges. Obviously, the scale can also be a microbiome habitat (body part) of an individual (e.g., gut, oral, skin, lung and vaginal of an individual); then metacommunity and metagenome assemblage can be defined for each individual or at individual level. Intuitively, I argue that the ecological theories for metacommunity can be extended to the metagenome assemblage, which is indeed a main objective of this article. Nonetheless, it should still be conscious with their difference: the basic measure in metacommunity is organismal taxon (e.g., species) abundances, while it is the metagenomic gene abundance in metagenome assemblage [9–11]. The assemblage of metagenomes is also different from the assemblage of gene mutations in population genetics because in the latter, all genes are from a single species, but in the former, it is the total genes carried by all species from a microbial community or a microbiome sample.

Metagenomic genes (MGs) are genes assembled and annotated from the whole-genome (shotgun) sequencing *reads* of microbiome samples, and the abundances of MGs can be organized as metagenomic gene abundance (MGA) tables. The MGA is essentially a matrix with each row of elements representing for the abundances of all MGs contained in a microbiome samples, and each column for the abundances of a certain MG (metagenomic gene) across all microbiome samples of the focal project. A fundamental difference between OTU and MGA tables is the “abundances of organisms or OTUs” versus “the abundances of genetic materials or MGs”. A second difference between the OTU table and MGA table is the magnitudes of the matrix elements. In general, the number of MGA matrix elements is 2–3 orders of magnitude larger than that of OTU (taxon) matrix elements. This seemingly nonessential difference turned out to have far reaching impacts on consequential data analysis and interpretations. The millions of MGs make the metagenomic data analyses such as bioinformatics, ecological and network analyses rather challenging. For example, performing complex network analysis with millions of nodes (MGs) is beyond the capacity of typical computational biology centers. In fact, the sheer size of MG numbers has been a major roadblock for the data analysis of whole-genome metagegnomic sequences. This has significantly delayed the applications of ecological and network analyses in the metagenome studies, and may explain the relatively few applications. To overcome the obstacle, Ma and Li (2018) proposed the concepts of MFGC (metagenome functional gene cluster), MF (metagenome function) and MP (metagenome pathway), which are similar to widely used CAG (co-abundant gene group) and MGS (metagenomic species, which are the CAGs with > 700 genes) but computed in terms of meta-transcriptomic functions or metabolomic pathways, rather than on statistical clustering based on gene abundances [9, 12, 13]. The introduction of MFGC/MF/MP readily removed the previously mentioned roadblock associated with bulk data of MGs because the numbers of MFGC/MF/MP are in hundreds or tens, which puts the computational load on a par with the OTU data analysis. Furthermore, transforming the MG to MFGC/MF/MP (i.e., the data reduction) is justified because (i) the function and pathway information is preserved, (ii) many of the millions of MGs are functionally redundant, (iii) there is currently little understanding of many individual MGs other than a mechanically assigned numeric gene number.

In previous studies [9–11, 14], we have demonstrated the applications of diversity, heterogeneity and their scaling analyses for metagenomic datasets. In the present study, I further attempt to investigate the critical ecological processes (mechanisms) underlying the metagenome patterns. As introduced previously, a primary theoretic tool to fulfill our objective is the extension of ecological neutral theory to the new domain of MG/MFGC, which has not been approached previously to the best of our knowledge. Since the neutral theory approach can only cover three of the four processes and in its ideal form, quantifying the effects of selection with neutral theory alone is obviously inadequate. That said, I adopt core/periphery network (CPN) [15, 16] and high-salience skeleton network (HSN) [16–18] to complement neutral-theoretic approach in order to assess and interpret the critical ecological processes underlying the metagenome patterns. Finally, I applied the stochasticity analysis framework to cross-verify the findings from neutral-theoretic and CPN/HSN network analyses [19].

*Selection* can be defined as deterministic fitness differences between individuals (or genes) of different species (or MFGCs) or the deterministic interactions among species (or MFGCs) and between species (or MFGCs) and their environments [6, 20]. In other words, *selection* represents asymmetric or unequal MFGC interactions from a network link perspective. From a network node perspective, selection represents heterogeneity or nestedness. While the CPN can classify all species as core and periphery network nodes (which have distinctively different interaction patterns), the HSN can identify high-salience skeletons (links) that form the backbone paths of species interactions, similar to distinguishing roads as highways (backbones for transportation) versus country roads in a transportation network. In other words, the distinction between core and periphery MFGCs is critical because CPN structures can effectively characterize complex sets of cooperative and competitive interactions between network nodes (MFGCs/MFs/MPs). Similarly, the HSN highlights the criticality of “highways” or “backbones”—network paths consisting of links with strong interactions. Given that nodes and edges (links) fully describe a network (model of metagenome), an integrated approach with CPN and HSN analyses can span the full spectrum of species interactions in driving the patterns of metagenome assemblages from both node and link perspectives.

**Fig. 1:**
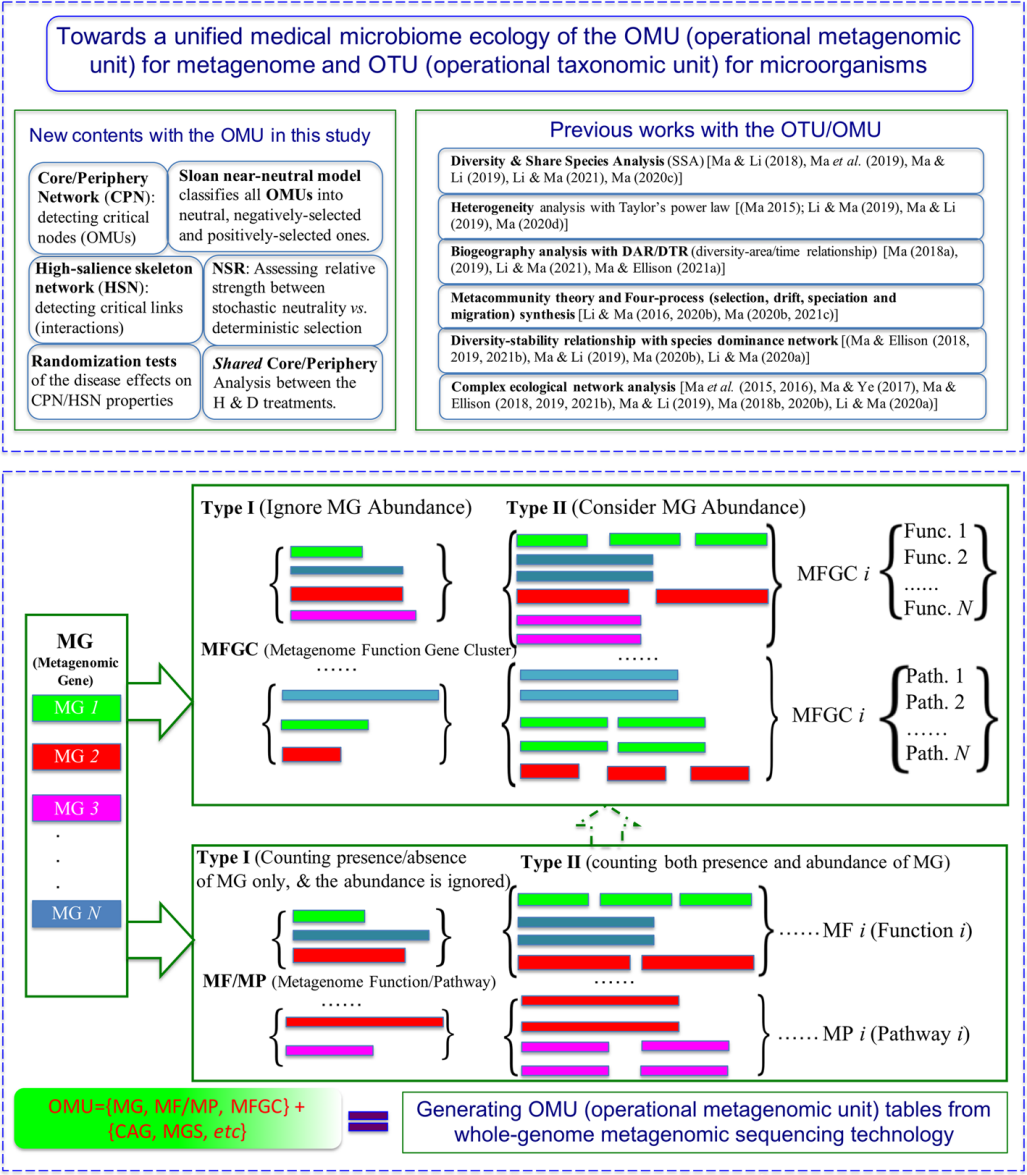
Diagramming the study design towards unified medical microbiome ecology of metagenomes (OMU) and organisms (OTU). The whole diagram consists of top section and bottom section, as well as formal set-theoretic (mathematical) definition of OMU, which are interpreted below. (**1**) The top section displays the study design consisting of two parts, i.e., the previous works (for the OTU and three topics of the OMU) including diversity, heterogeneity and biogeography) and the contents planned for this study. The new contents with the OMU in this study include six approaches: two network-approaches with core/periphery network (CPN) and high-salience skeleton network (HSN), Sloan near-neutral model, normalized stochasticity ratio (NSR), two statistical test approaches (randomization tests and shared OMU analysis) for detecting the disease effects. (**2**) In the bottom section, the *ad-hoc* concept OMU (operational metagenomic unit) or its shorthand MU is introduced as the counterpart of OTU (*see* the methods section for their interpretations), and MG (metagenomic gene) is considered as the basic (‘atomic’) unit of OMU and is similar to (the counterpart of) the *species* in the OTU (97% similarity in 16S-rRNA sequences for bacteria) taxonomic hierarchy. Both MGs and species exist as basic (undividable) units. In the case of MG, each MG may have one or more functions, but the ‘components’ (if forced to divide) of MG is sequencing reads that do not have a corresponding function (hence being atomic). Species is the foundation of a taxonomic system in the case of OTU hierarchy. The other entities of OMU include MF/MP/MFGC (defined by Ma & Li 2018: *Mol. Ecol. Res*.) and CAG and MGS (by Li et al. 2014; Nielsen et al. 2014, both in *Nature Biotechnology*). All of them are generated from MGs, just like other taxonomic units such as genus and family are combinations of species. (**3**) Formal mathematical definitions for MF/MP/MFGC from the MG are defined as follows. Assuming there are *n* MGs, i.e., MG*1*, MG*2*,…, MG*n*, we can define MF/MP/MFGC with mathematic set notation: $MF = \left\{ {MG1, \, MG2, \, MG3, \ldots } \right\}$, where *MG1, MG2, MG3* are mapped to same metagenomic function. MP can be defined similarly except that all of its genes (MGs) are mapped to same metagenomic pathway. Therefore, MF (or MP) can be described as a set of the MGs annotated to the same metagenomic function (or pathway). Conceptually, MFGC is a set of subsets of MFs (or MPs). That is, the elements of MFGC set consist of the combination of MFs. For example, $MFGC = \{ \{ MF1, \, MF7\} \}$, this MFGC consists of the MGs that simultaneously annotated to two metagenomic functions *MF1*, and *MF7*. Assuming there are *m* possible MFs, the total possible number of MFGC is equal to $M = C_{m}^{2} + C_{m}^{3} \cdots + C_{m}^{m - 1} + C_{m}^{m}$. In practice, only a tiny portion of the possible number exists naturally

Furthermore, if our integrated neutral-theoretic and network approaches to the patterns of metagenome assemblages (i.e., the counterpart of metacommunity in the community ecology) are successful, we are a step closer to a unifying microbiome ecology of metagenomes and taxa. Figure 1 illustrates the study design of this paper, including the ecological and network approaches towards a unified microbiome ecology of metagenomes and organisms (species, taxa, or OTUs).

Justification for unifying community ecology of organisms and metagenomes, especially from functional perspective, are also supported by Boon et al. [21] comprehensive review. They suggested that the units for delineating biodiversity can be taxonomic, phylogenetic, or functional in nature, since microbes could rapidly gain and lose genes, likely delinking community roles from taxonomic and phylogenetic clustering [21]. Instead, Boon et al. [21] recommended that trait-based (function-based) methods provide a useful substitute because many traits can be defined in terms of gene functions, metabolic modules, and genomic properties, similar to MF/MP/MFGC defined in our study. Boon et al. [21] further argued that an analysis that considers both taxon assignment and traits in concert should be preferred, since they may complement each other. They also suggested that individual genes, similar to metagenomic genes (MGs) used in our study, also deserve consideration as units (entities) in ecological analyses, assessing features including diversity, turnover, and interactions modeled using genes rather than organisms as entities. In the present study, I translate the ecological and network approaches we previously developed for analyzing microbial communities using taxon entities such as microbial species or operational taxonomic units (OTUs) for the metagenome analysis.

Unifying the community ecology of organisms and metagenomes is also challenging. First, the applicability of ecological theories to assemblages of metagenomes should be self-evident as demonstrated by: (i) Interactions of metagenomic units (entities) such as MG/MFGC/MF/MP can be modeled with classic ecological theories [9–11, 22]. (ii) Classic community ecology is mainly about the studies of the interactions within and/or among assemblages of species (community/metacommunity) and their interactions with the environment, formally the spatiotemporal dynamics of community (metacommunity) at local, regional and global scales influenced by the environment, as well as their impacts to the environment. In the case of human microbiomes and metagenomes, environment is the human body or microbiome host. In fact, with the unifying community ecology of metagenomes and organisms or OTUs, we are equally, if not more, interested in the impacts of metagenomes and their carriers (microbiomes) on their environment (human hosts). The unifying community ecology of metagenome and organisms can be considered as part of the emerging medical ecology of microbiome-associated diseases, which is an cross-disciplinary field of medical microbiology, computational biology and bioinformatics (for supporting metagenomic technologies), and theoretical ecology for modeling and simulating of microbiomes [20, 23]. However, the cross-disciplinary nature of medical ecology also presents significant challenges such as establishing common terminologies, identifying appropriate theories, and crossing possible technological and conceptual barriers. I hope that the ecological methods demonstrated with metagenomes of human microbiomes in this study can be a small step towards a unified ecology of metagenomes and organisms, or of the microbiomes and macrobiomes with gene and species in general.

## Datasets and methods

### Seven metagenome datasets of the human gut microbiome

This subsection briefly introduces the datasets I use to demonstrate my efforts for unifying the microbiome ecology analyses of the OMU (operational metagenomic unit) for metagenomes and the OTU for microorganisms. All of the seven reanalyzed datasets of human gut metagenomes are already available in public domain and were published previously by other researchers (Table 1). To keep balanced sample sizes between the healthy and diseased treatments, in two datasets, I randomly discarded certain number of samples, so that the results between the healthy and diseased treatments can be compared properly. A brief introduction on the metagenome entities including MG (metagenomic gene), Type-I and II MFGC (metagenome functional gene cluster), Type-I and II MF (metagenomic function, based on eggNOG database), and Type-I and II MP (metagenomic pathway, based on KEGG database) is illustrated in Fig. 1. The difference between Type-I and Type-II is that the former ignores the abundance of metagenomic genes and only counts the presence/absence of MG (*see* Ma and Li 2018 for detailed definitions) [9]. The terminology of Type-I MFGC (MFGC-I) and Type-II (MFGC-II) were previously defined by Ma and Li [9]. To make both terms more self-explaining, I suggest giving Type-I MFGC an alias of “non-abundance-based (non-abundance) MFGC” and Type-II MFGC as alias of “abundance-based MFGC.”

**Table 1:** Summary information on the seven datasets of the human gut metagenome reanalyzed in this study as well as the numbers of OMUs (operational metagenomic units)

Study	Treatment	Number of samples	Number of samples used in this study	Number of MFGCs (eggNOG)	Number of MFGCs (KEGG)	Number of MP/MF (eggNOG)	Number of MPF (KEGG)	Number of MGs	Reference (data source)
Stool (HMP)	Stool (Healthy)	139	139	361	312	37	24	5838354	Methé et al. [24]
Obesity	Lean	96	96	361	312	37	24	5407291	Qin et al. [25], Chatelier et al. [26]
	Obesity	17	0	0	0	0	0	0	
	Overweight	168	96 Sampled from 168	361	312	37	24	5134721	
Type-2 Diabetes	Healthy	74	74	361	312	37	24	4573927	Qin et al. [27]
	Disease	71	71	361	312	37	24	4432814	
IBD (Inflammatory bowel disease)	Healthy	24	71	361	312	37	24	2898618	Nielsen et al. [13]
	Healthy Relative	47							
	CD	21	0	0	0	0	0	0	
	UC	127	71 Sampled from 127	361	312	37	24	4462890	
Total		784	618						

As a side note, the computational load for the metagenomic analysis of the whole-genome sequencing data is usually far more demanding than that for the marker-gene (e.g.,* 1*6S-rRNA) based analysis. The computational work of this study was performed on a server containing double CPU (Intel Xeon Silver 4114 CPU, each with 12 cores) and 512 GB-memory space.

### A brief review on the metagenomic terminology

This subsection briefly reviews and summarizes existing, relevant concepts/terminologies for metagenome studies, and further suggests the usage of operational metagenomic unit (OMU) for classifying and cataloguing metagenomic genes as a counterpart concept of the familiar operational taxonomic unit (OTU) for the taxonomic classification of microbes. It is well known that the classification of microbial OTUs is usually based on marker-gene (e.g., 16S-rRNA) sequencing technology, while the OMU is suggested as a concept for supporting the whole-genome sequencing technology, which produces total genes (collective genomes) contained in a environmental microbiome sample.

Unlike in the fields of amplicon-sequencing (e.g., 16S-rRNA) data processing and/or other fields of microbiology such as microbial taxonomy and ecology, there is not yet a well accepted terminology system that covers all commonly used terms in studies on metagenomes. While some concepts, most notably, metagenome, and to a slightly less extent, metagenomic gene (MG), transpire unambiguous meanings to virtually all microbiologists and even most biologists and ecologists, some other concepts such as IGC (integrated gene catalog), MGS (metagenomic gene species), and CGA (co-abundance gene group) may still be familiar to most microbiologists, and still some concepts such as ORF (open reading frames), orthologous groups (KEGG, eggNOG, etc.), MFGC (metagenome functional gene cluster), MF (metagenome function), MP (metagenome pathway) may only be familiar to specialized fields in microbiology and informatics. Yet, researchers still occasionally coin new ad hoc terminologies that may only be known to readers of their respective papers.

As illustrated in Fig. 1 and mentioned previously, I suggest using the term “operational metagenomic unit” (OMU) to mirror the term OTU (operational taxonomic unit) that is obviously on a par with metagenome. However, there are not totally reciprocal. First, unlike OTU, the items I wished to cover with OMU (as listed previously) are not necessarily hierarchical, not necessarily homogenous within the same hierarchical level, and instead, are mostly “networked” (cross-linked) and heterogeneous. Second, unlike OTU that transpires the message of imprecision (or workaround) in classifying microbes to mirror the taxa of macrobes (plants and animals), most entities or items in my provisional OMU are not workaround solutions as rightly advised by an anonymous expert reviewer. I also suggest to simply use the term “metagenomic unit (MU)”, as shorthand for and interchangeably with, the term OMU. That is, I use the term MU or OMU as a collective proxy for multiple metagenomic entities defined and/or used in our previous publications and also analyzed in this study, including metagenomic gene (MG), MFGC (metagenome functional gene cluster), MF (metagenome function) and MP (metagenome pathway) [9–11].

The metagenome refers to the “collective genome” of an environmental sample. Note that the term ‘environmental’ is used broadly to cover virtually any place on the earth planet (assuming that life only exists on the earth). While the genome of a species can be considered as a catalog of all genes the species carries, metagenome of an environmental sample (such as a sample of human gut microbiome) can be considered as a catalog of all genome the environmental sample contains. Following this convention, Li et al. [12] used the term “integrated gene catalog” (IGC), but they did not define a formal term for the content or items of the IGC. Ma and Li [9] introduced the term “*metagenomic gene*” (MG) to refer to the items or content of IGC. Ma and Li [9] noted: “metagenome assembly generates millions of *contigs*, which are fed into *gene prediction* software, and the latter generates a list of nonredundant genes based on the criteria set by ORFs (open reading frames). MG embodies single‐gene‐level genetic information.” Therefore, MGs of a metagenome can be considered as the counterpart of genes in a genome, which are also similar to (or being the counterpart of) the species-level unit in the OTU (operational taxonomic unit) for the taxonomy (organismal classifications) of microbes.

In Li et al. (2014), they further annotated and binned the genes (MGs in terms of Ma and Li 2018) in IGC based on KEGG and eggNOG databases as so-termed *orthologous groups* [9, 12]. The term *orthologous group* was not a new term in Li et al. (2014); instead it is a term native to functional KEGG and eggNOG databases [12]. So in their paper, they used KEGG *orthologous group* and eggNOG *orthologous group*, respectively. Both KEGG and eggNOG are essentially catalogs (databases) of *functional genes* because their contents are obtained from grouping genes according to their *functions* or *functional categories*. In consideration of lack of common terminologies, and also KEGG/eggNOG contains both genomic and metagenomic genes, Ma and Li (2018) introduced the terms of MFGC (metagenome functional gene cluster), MF (metagenome function), MP (metagenome pathway) to deal with the lack of a consistent terminology, all of which are clusters of MGs (metagenomic genes) based on their metagenomic functions or function category [9]. MFGC was defined based on metagenome functional gene cluster (category), and MF/MG was defined based on kinds of metagenome functions or pathways. Their computational steps were introduced in Ma and Li [9]. Figure 1 further illustrated the relationships among MG, MF/MP and MFGC, including the set theoretic definitions for MF/MP/MFGC. Mathematically, a MF (or MP) is a set with MGs as its elements, and a MFGC is a set with subsets of MFs as its elements, as illustrated in Fig. 1.

Beyond Li et al.(2014) ICG [12], there have been multiple algorithms [13, 28–30] to produce IGC-like catalogs described by various terms such as “metagenomic gene catalogs” or “metagenomic assembled genomes” (MAG), all of which can be considered as basic or ‘atomic’ components of metagenome. But the underlying principle and end results (catalog or list of genes) can be adequately captured by the concept of MG of Ma and Li [9]. A commonality here is the basic or atomic nature of the metagenomic entities (the MG and other similar ones), under which the divisible components such as contigs and reads are *not* mapped to any functions. For this reason, I stick to the term MG in this study and suggest following it in future literature.

Similar to MF/MP and MFGC, there are other similar metagenomic entities (units) such as CAG (co-abundance gene group) and MGS (metagenomic species) [12, 13]. Nielsen et al.[13] developed a sophisticated algorithm to cluster metagenomic genes into CAGs, and the algorithm was primarily based on Pearson’s correlation coefficient (> 0.9) between a randomly selected ‘seed’ and the other genes with similar abundance profiles. With their algorithms, many MGs can be clustered into different CAGs, but some MGs may not be classified into any CAGs. Nielsen et al.[13] further referred to these co-abundance gene groups (CAGs) as metagenomic species (MGS) if they satisfy two conditions: (i) with more than 700 metagenomic genes; (ii) if more than 50% of the genes comprising the CAG were assigned a given microbial species level taxonomy (including genes with no match). Again, only portions of MGs could be mapped to MGS. It was for this incomplete nature of mapping from MGs to high-level units including CAGs and MGSs, that prompted Ma and Li (2018) to introduce the concept of MF (MP) to represent the MG set of functional (pathway) genes, and each MF (MP) contains all the genes (MGs) of the same function (or pathway), i.e., a set of genes (MGs) with the same function or pathway [9]. Similarly, they introduced the concept of MFGC to represent the MG sets of a functional gene cluster (category), and each MFGC contains all genes of same function (pathway) cluster. Each cluster of functions (pathways) may contain two or more functions or pathways; hence, each MFGC contains all of MGs annotated to the functions (pathways) belonging to same cluster. By mapping against major function/pathway databases (such as KEGG and eggNOG), all MGs can be mapped to MF (or MP) and MFGC.

From above introduction, it is clear that we currently still lack a unified or consistent terminology system to cover all of the terms mentioned previously. As suggested previously and interpreted in Fig. 1, I use the term OMU (operational metagenomic unit) or MU (metagenomic unit) for short, to cover all of the metagenomic entities (units) including MGs, MFGC, MF/MP, CAG, MGS, etc.in this article as a shorthand or workaround term. Among the items covered by OMU, MG can be considered as the basic (atomic) unit in the OMU hierarchy (more accurately “tree” or “network”, i.e., some terms are cross-linked to form a tree or network structure among the terms), since MGs are obtained from metagenomic contigs and contigs do not have explicit mapping to functions (Fig. 1). Furthermore, the other terms such as MFGC, MF/MP, and MGS are clusters of MGs based on specific algorithms designed to map MGs to functions/pathways or groups of functions/pathways [9].

In summary, the previous conceptual and terminological discussion is aimed to facilitate the applications of ecological and network approaches to the analysis of metagenomic sequencing data. Previously, these approaches (as introduced below) have been successfully introduced to the analysis of amplicon-sequencing data, which are first processed by special bioinformatics pipelines to generate the OTU tables. It is the OTU tables that are the input data for the ecological and network analyses. Similarly, the whole-genome metagenomic sequencing data are first processed by special bioinformatics pipelines to produce MG tables or other OMU (MFGC, MF/MP, etc.) tables [9, 12, 13, 28, 29]. It is the OMU tables that are the input data for the proposed ecological and network analyses, similar to the input of OTU tables for the ecology of microbiomes (microorganisms) [9–11, 31–41]. In spite of the differences between OMU and OTU (genes vs. organisms) explained previously, as demonstrated in the remainder of this article and also previously [9–11, 31–41], those ecological/network approaches seem to be equally powerful in producing biomedical insights, which encourages us to advocate for a unified medical ecology of metagenomes and organisms.

### Sloan (2006, 2007) near neutral theory model

The near neutral theory model introduced in this subsection is designed for studying the community assembly and diversity maintenance of organisms including microorganisms. The neutral theory for biodiversity is inspired by the neutral theory for molecular evolution, but the theory has not been applied to metagenomes, to the best of my knowledge. Here, we propose to apply the neutral theory of biodiversity, specifically Sloan’s near neutral model, for metagenomes at various OMU scales. The differences between Sloan et al*.* [42, 43] near neutral model and Hubbell [3] general unified neutral theory of biodiversity (UNTB) lie in: (i) With Sloan model, a species is allowed to possess competitive advantage (above-neutral) or disadvantage (below-neutral). For this, it is a near neutral model. According to Sloan model, all species in metacommunity can be classified into three categories: neutral, below-neutral and above-neutral. (ii) Sloan model is formulated as a continuous *diffusion* equation, which allows to model nearly arbitrary size of populations (communities) that are usually significantly larger than the sizes of macrobial populations (communities) of plants and animals. This is actually the primary reason why Sloan model is selected for this study because of the huge sizes of metagenomic genes (MGs), which are in millions and could not be fitted with standard UNTB models because of the limitation of standard software algorithm for the UNTB. (iii) Sloan model has a built-in mechanism to adapt to (calibrate and validate) a frequently occurring scale disparity in metagenomic sampling between the number of genetic sequences that are analyzed and the number of individuals (or metagenomic genes in the case of this application) in communities (or metagenomes in this application). Sloan et al*.* [43] presented an example of the scale disparity: a 10-*g* sample of soil may contain 10^10^ individuals of microbes (the approximate world human population), but the individuals (genetic sequences) identified by 16s-rRNA sequencing are much fewer (hundreds to thousands). For the detailed model derivation and computation procedure of Sloan model, readers are referred to Sloan et al. [42, 43]. A brief introduction on Sloan model is provided below.

Examples of applying Sloan models to 16s-rRNA sequencing reads from amplicon sequencing (i.e., for studying neutrality of microbial communities) include Venkataraman et al.[44], Burns et al. [45], Li et al.[46], Sieber et al. [47]. To the best of our knowledge, this study should be the first application of Sloan model to metagenomic genes (metagenomes) obtained via whole-genome (shotgun) sequencing technology. In this study, in places of microbial taxa, Sloan model is applied to the MGs (metagenomic genes) and Type-II or abundance-based MFGC (metagenome functional gene cluster, in which both the kinds of MGs and their abundances are taken into considerations for the Type-II). As a side note, Sloan model cannot be applied to Type-I MFGC nor to MF/MP (metagenomic functions)/(metagenomic pathway) due to their data structures (i.e., gene abundance is ignored and only the presence/absence of the MG is counted).

Sloan et al. [42, 43] model represented the metacommunity as consisting of source and local communities. In Sloan model, the local community is saturated with *I*_*T*_ OTUs (MUs in this article), suggesting that any death (disappearance) of OTUs (MUs) will be replaced by either local reproduction (mutation) or remote immigrations (carried by remote individuals). It assumes that the probability of from remote immigration is *m*, and from offspring of local reproduction is (1-*m*).

Sloan et al*.* [42, 43] derived the probability that the abundance of the *i-*th OMU is increased by one, decreased by one, or is unchanged, is as follows, respectively:

1 $$\Pr (I_{i} + 1/I_{i} ) = \left( {1 - \frac{{I_{i} }}{{I_{T} }}} \right)\left[ {mp_{i} + (1 - m)\left( {\frac{{I_{i} }}{{I_{T} - 1}}} \right)} \right]$$

2 $$\Pr (I_{i} - 1/I_{i} ) = \frac{{I_{i} }}{{I_{T} }}\left[ {m(1 - p_{i} ) + (1 - m)\left( {\frac{{I_{T} - I_{i} }}{{I_{T} - 1}}} \right)} \right]$$

3 $$\Pr (I_{i} /I_{i} ) = \frac{{I_{i} }}{{I_{T} }}\left[ {mp_{i} + (1 - m)\left( {\frac{{I_{i} - 1}}{{I_{T} - 1}}} \right)} \right] + \left( {\frac{{I_{T} - I_{i} }}{{I_{T} }}} \right)\left[ {m(1 - p_{i} ) + (1 - m)\left( {\frac{{I_{T} - I_{i} - 1}}{{I_{T} - 1}}} \right)} \right]$$

where *p*_*i*_ represent the occurrence frequency of the *i-*th OMU in the source community and *I*_*i*_ represent the abundance of *i-*th OMU in the local community. Let *x*_*i*_ = *I*_*i*_/*I*_*T*_ be the occurrence frequency of the *i-*th OMU in the local community. The predicted abundance (*ϕ*_*i*_) of the *i*-th OMU follows the beta distribution:

4 $$\phi_{i} = cx_{i}^{{I_{T} mp_{i} - 1}} (1 - x_{i} )^{{I_{T} m(1 - p_{i} ) - 1}}$$

where *c* is a complex gamma function of (*I*_*T*_, *m* and *p*_i_), see Sloan et al. [42, 43] for its form.

Burns et al*.* [45] summarized the process for testing Sloan’s neutral model as follows:

[Step #*1*] Calculate *p*_*i*_ and *x*_*i*_, fit beta distribution and obtain the estimate of *m*.

[Step #*2*] Calculate the theoretical occurrence frequency of *i*-th OMU across all local communities (samples) with* m* and the beta distribution.

[Step #*3*] Determine whether or not the observed *x*_*i*_ of OMU *i* falls within its 95% confidence interval predicted from Sloan model, and further classify each OMU as neutral, above-neutral, or below-neutral.

### The core/periphery network (CPN)

Ecological communities/systems are typical complex systems, which can be modeled with complex networks including the so-termed core/periphery network (CPN). Previously, the CPN was applied to analyze species or OTU correlation (co-occurrence) networks based on amplicon sequencing reads. In this sub-section, I suggest to apply the CPN for building and analyzing OMU correlation (co-occurrence) networks. The concept of core/periphery in ecological communities (systems) can be traced back to 1960s. For example, Margalef [48] identified the role of asymmetry and heterogeneity in ecological communities. Robert May’s [49] classic work first proposed that network stability can be explained either by a nested-like *core/periphery* structure, or by *network modules*. Theoretical and simulation studies have revealed that network *cores* enhance system *robustness* and *evolvability*, which render system to adapt to large environmental perturbations, as well as to noise from intrinsic processes [15, 16, 50].

One way to define core/periphery network is to answer the question: what is a perfect or ideal core/periphery network? A perfect or ideal core/periphery network is composed of a fully linked core and a periphery that is fully connected to the core; however, none of the periphery nodes are connected with each other (Csermely et al. 2013). Such a perfect or ideal CPN rarely exists in practice. For example, in a real world CPN, there are sparse links between periphery nodes, and a core is not necessarily a clique (i.e., fully connected).

Specifically, assume *G* = (*N*, *E*) be an undirected, unweighted graph with *n* nodes and *m* edges, and let *A* = (*a*_*ij*_) be the adjacency matrix of graph *G*, where *a*_*ij*_ = 1 if there is an edge between node *i* and node *j* and *a*_*ij*_ = 0 if not. Let *Δ* be a vector of length *n* with elements of 1 or 0, assuming that the corresponding node belongs to the core or the periphery. Furthermore, let *P* = (*p*_*ij*_) be the adjacency matrix of the ideal or perfect core/periphery network with *n* nodes and *m* edges. The determination of core-periphery structure is an optimization process to discover the vector *Δ* so that the objective function (*ρ*) attains its maximum. With the vector *Δ,* it is then simple to classify nodes into either core or periphery.

5 $$\rho = \sum\limits_{i,j} {A_{ij} } P_{ij}$$

However, it is infeasible to build a CPN with MGs (which are in millions) on a typical computational platform); I instead build CPN for the upper level OMUs, i.e., the MFGC and MF/MP units. When building the CPN with MFGC (MF/MP) as nodes and their interactions as links, the CPN structures reflect the heterogeneity or asymmetry (non-equivalence) of functional gene clusters, protein functions or metabolomic paths from node perspective. In terms of the four-process synthesis for community ecology and biogeography [6–8], heterogeneity or asymmetry of OMU nodes can represent the effects of *selection,* and the CPN offers a tool to assess and interpret the *selection* effects from the node perspective, which can be cross-verified by the HSN (high-salience network) analysis from the link (edge) perspective explained below, and also be cross-verified by the previous neutral theory modeling as well as the stochasticity analysis framework by Ning et al. [19].

When building and analyzing the CPNs, all the network nodes with MUs are automatically categorized into either the core or periphery, which have contrastingly different interaction patterns as explained previously and can be of critically different ecological roles or biomedical implications in the case of microbiome associated diseases (obesity, type-2 diabetes, and IBD). I consider this distinction between core and periphery nodes as originated from possible selection effects, which may be evolved on evolutionary time scale and may be shaped by environmental disturbances such as disease effects on ecological time scale. I perform two kinds of randomization tests to determine the disease effects: (i) Test whether or not the *shared* core/periphery nodes (OMUs) between the healthy and diseased metagenome treatments rise or decline (more or less than by chance) due to the disease effects. (ii) Test the differences between the healthy and diseased treatments in their CPN properties such as strengthen of core, nestedness, and ratio of core to periphery nodes.

### The high-salience skeleton network (HSN)

While the previously described CPN distinguishes the different structural and functional roles between core and periphery nodes (OTU or OMU), the high-salience skeleton networks (HSN) makes distinctions among the links (edges) among OTUs or OMUs. That is, similar to the previous suggestion to use CPN for building OMU correlation networks, and here I suggest applying the HSN for building and analyzing OMU correlation networks.

The capacity in reducing the *complexity* of complex systems (networks) but preserving their certain key features is considered as a primary reason (also an advantage) why network analysis has been experiencing explosive applications since the start of the twenty-first century. Network analysis can shed critical insights on complex systems such as ecological communities by reducing complexity and leveraging the insights from the key features. The HSN allows one to focus on critical paths (interactions), known as backbones, in complex networks, while the previous CPN allows for focusing on the differentiations of nodes (core vs. periphery) [16–18].

High salience skeletons or backbones effectively reduce the number of links in the network while preserving the nodes [17, 18]. However, reducing link complexity can be particularly challenging due to the inter-dependence of link and node heterogeneity. To address the challenge, Grady et al. [17] introduced the concept of *link salience*, which measures the importance of a link by considering the “votes” of an ensemble of nodes on the link’s importance to the network. The link salience then quantifies the level of ‘consensus’ that exists among nodes regarding the link’s importance. Using an analogy in a transportation network, the importance of a highway is ‘voted’ by the residents of the cities (nodes) in the transportation network, based on their experiences. The so-termed high-salience skeletons then constitute the *backbones* (similar to inter-state “highways”) of the network, which are much more important than state highways or rural roads. Grady et al*.* [18] argued that the emergence of backbones should be attributed to the interplay of broadly distributed node degrees and link weights. Therefore, the HSN eliminates large number of less important interactions (links) without sacrificing the heterogeneity information carried by nodes; using an analogy, voters from different cities (network nodes) with different backgrounds (such as road usages) collectively decide which highways are critical for the whole transportation network, and which roads are not essential.

According to Grady et al. [17], link salience (*s*) is defined according to the notion of shortest paths in weighted networks. For the species correlation network, the *inverse* of correlation coefficients can be used as weights [16]. With this scheme, the links with closer correlation relationships are counted more in voting for high salience links. With a weighted network defined by weight matrix *w*_*ij*_ and a shortest path between node *x* and *y*, the indicator function is defined as:$\sigma_{ij} (y, \, x) = 1$ if edge (*i*, *j*) is on the shortest path from *x* to *y*, $\sigma_{ij} (y, \, x) = 0$, otherwise. A shortest path tree *T*(*x*) rooted at node *x* is defined by a matrix with elements: $T_{ij} (x) = 1$, if $\sum\limits_{y} {\sigma_{ij} (y, \, x) > 0}$, $T_{ij} (x) = 0$ otherwise. Link salience *s*_*ij*_ of edge (*i, j*) is defined with the following equation:

6 $$s_{ij} = \frac{1}{N}\sum\limits_{x} {T_{ij} } (x) = \left\langle {T_{ij} (x)} \right\rangle_{V}$$

where $\left\langle \cdot \right\rangle_{V}$ is the average across the set of root nodes *x*.

In the case of human metagenomes, the high salience skeletons or backbones reflect the heterogeneity or asymmetry of OMU (MFGC/MF/MP) interactions from the link perspective. According to the four-process synthesis [6–8], heterogeneity (or asymmetry) of OMU interactions or the OMU’s interactions with their environments can reflect the effects of *selection,* which is evaluated with the HSN, cross-verified with the previously introduced CPN, Sloan near neutral model, as well as the stochasticity analysis framework [19]. Similar to previous CPN analysis, I also test the disease effects on the HSN properties such as the proportions of high-salience links, and network assortativity.

### Special trio motifs and P/N ratios in metagenome networks

As mentioned previously, May’s [49] seminal work had already suggested that network stability can be achieved either by the development of a nested-like core/periphery structure, or by network modules. Though there are numerous methods for determining and characterizing network modules, it appears that there is not yet a widely recognized guideline on which method may be the most appropriate for identifying modules. In the present study, I adopt the principle of parsimony, using arguably the simplest module—special trio motifs detection method by Ma and Ye [51]. A justification for focusing on trio motifs is that the trios are fundamental building blocks for arguably all network modules. Ma and Ye [51] defined *15* special trio motifs and they are special because one of their nodes must be MAO (most abundant OTU) or MDO (most dominant OTU). In this study, the MAO is replaced with the most abundant OMU (MFGC or MF/MP). Another feature of the 15 trio motifs is the consideration of the interaction type (cooperative vs. competitive interactions, *or* positive vs. negative). I will test whether or not diseases have significant influences on the occurrences of the special trio motifs in human gut metagenome networks. Besides special trio motifs, I also use the P/N ratio—the ratio of *positive* to *negative* links—for assessing the balance between the cooperative (positive) interactions and competitive (negative) interactions in complex networks, including CPNs and HSNs [52]. Both trio-motifs and PN ratio may act as in silico biomarkers for measuring the effects of diseases on the interactions between OMUs, just as their previous applications to the complex networks of OTUs.

### Integration of neutral-theoretic modeling and core/periphery networks for OMU analysis

As mentioned previously, Sloan [42, 43] near neutral model, like Hubbell [3] classic neutral model, covers three (drift, dispersal, and speciation) of the four processes (mechanisms), excluding selection, which shape the community assembly and diversity maintenance. In contrast, the core/periphery network can capture the asymmetrical (non-equivalent) aspects of the metacommunity (each metacommunity forms a complex network in the form of CPN or HSN) from both nodes (OMUs) and edges (interactions between OMUs) perspectives. Given that the non-equivalence (asymmetry) is the opposite of equivalence among OMUs, the hallmark of neutral theory, I conceive that CPN/HSN can be complementary to neutral-theoretic analysis. Nevertheless, I caution that this complementary hypothesis between neutral theory and CPN/HSN approaches to the four-processes synthesis is largely conceptual, rather than quantitative [6–8]. Therefore, I can neither claim that the metrics of neutral theory and CPN/HSN may fully interpret the community dynamics, nor partition the dimensions of the four processes.

While the above-described complementary approach does not need direct integration of both the approaches and is largely conceptual and qualitative, still the neutral-theoretic and network approaches can be integrated directly. A simple integration scheme can be to apply Sloan model to the core and periphery nodes, respectively, and then check if the core and periphery structures have different neutrality/selection (the complementary force of neutrality) levels. The following simple two-step processes implement this integration:

[Step #1] Apply the previously outlined procedures (e.g., Eq. 5) for building the CPN to the human metagenome datasets, and obtain lists of core and periphery OMUs, respectively. Essentially, we get two OMU tables, one for the core and another for the periphery.

[Step #2] Apply Sloan model to the OMU tables for the core and periphery respectively, and obtain two new lists, one for the core and the other for periphery. In each list, three kinds of OMUs, neutral, above-neutral, and below-neutral, are distinguished, just like in the case of standard Sloan model application, but, in which the whole metacommunity without distinguishing between core and periphery nodes (OMUs).

### Ning et al.(2019) stochasticity framework for estimating the relative strength of stochastic neutral drifts versus deterministic niche selections

Similar to the previously introduced ecological/network approaches that can be harnessed for both OTU/OMU analyses, the stochasticity framework of Ning et al. (2019) also provides a tool for analyzing the interactions among OMUs in driving the structures and dynamics of metagenomes. It has been suggested that the neutral theory may overestimate the strength of neutral processes, and various remedy approaches have been proposed to address the potential issues [53, 54]. To cross-verify the findings from Sloan near neutral model, and also to gauge the relative strength of stochastic versus deterministic forces in shaping the community assembly, I adopted Ning et al. [19] normalized stochasticity ratio (NSR) as an alternative approach to gauging the “low bounds” of the stochasticity level.

Ning et al. [19] maintained that deterministic processes should drive ecological communities more similar or dissimilar than null expectation, and they established a sophisticated framework to implement a null model for quantifying stochasticity. One important metric they used to develop their framework was the utilization of Ružička similarity metrics, which is defined based on species abundance [55]. Assume *C*_*ij*_ be the observed similarity between the *i-*th and *j-*th community,

7 $$C_{ij} = \frac{{\sum\nolimits_{S} {\min (p_{k}^{i} ,p_{k}^{j} )} }}{{\sum\nolimits_{S} {\max (p_{k}^{i} ,p_{k}^{j} )} }}$$

where *S* is the number of species, $p_{k}^{i}$ and $p_{k}^{j}$ are the relative abundance of *k-*th species in the *i-*th and *j-*th community.

Suppose there exist *m* local communities in a metacommunity, *C*_*ij*_ is the observed similarity between the *i-*th local community and the *j-*th local community in the metacommunity. *E*_*ij*_ is the null expected similarity between the *i-*th community and the *j-*th community in one simulated metacommunity. $\overline{{E_{ij} }}$ is for the average of the null expected similarity between the *i-*th and the *j-*th communities from 1000 simulated metacommunities. There are two possible ways to evaluate the community stochasticity: One is the deterministic processes that drive communities more similar, in which *C*_*ij*_ > $\overline{{E_{ij} }}$, and the stochasticity ratio (SR) from the first way (i.e., type A *SR*) is

8 $$SR_{ij}^{A} = \frac{{\overline{{E_{ij} }} }}{{C_{ij} }}$$

Another way is that deterministic processes drive communities more dissimilar, in which *C*_*ij*_ < $\overline{{E_{ij} }}$, and the stochasticity ratio (i.e., type B *SR*) is

9 $$SR_{ij}^{B} = \frac{{1 - \overline{{E_{ij} }} }}{{1 - C_{ij} }}$$

The actual stochasticity ratio in the whole metacommunity should be the weighted average of type A and type B SR, that is,

10 $$SR = \frac{{\sum\nolimits_{ij}^{{n^{A} }} {SR_{ij}^{A} + \sum\nolimits_{ij}^{{n^{B} }} {SR_{ij}^{B} } } }}{{n^{A} + n^{B} }}$$

in which *n*^*A*^ is the number of the pair-wise similarities that exceed null expectation, and *n*^*B*^ is the number of the pair-wise similarities that do not exceed null expectation. Therefore, *SR* reflects the *strength of stochasticity* in the community assembly, and should range from 0 to 100%. When the community assembly is totally deterministic without any stochasticity, then *SR* would be 0%; otherwise *SR* would be 100%. Ning et al*.* [19] further argued that if expected stochasticity is very low, *SR* could overestimate stochasticity. To remedy this potential issue, they developed a procedure to normalize SR to the range between 0 and 1. The normalized stochasticity ratio (*NSR*) was found to possess higher precision than the *SR*, and its adjustment from *SR* is referred to Ning et al.[19] to avoid complex formulae here. Nevertheless, in the present article, I do use the *NSR* to cross-verify the results from Sloan model. Similar to Sloan [42, 43] near-neutral model that designates source and destination communities, Ning et al.[19] normalized stochasticity ratio (*NSR*) measures the stochasticity (stochastic drifts) between (pair-wise) two communities by measuring their similarity. However, in the former, two groups of communities are designated, and in the latter, only two communities are compared. For this reason, directly comparing the results from Sloan model and NSR is not feasible, and still we can make comparisons between the percentages of neutral species (or OMUs) and NSR. Both the metrics should have the same trend of change patterns, although their absolute values may not be comparable.

### Computational and statistical procedures for CPN/HSN/Trios/PN-ratio analyses

Spearman’s correlation coefficients with FDR (false discovery rate) adjustment (*p*-value = 0.05) are first used to construct standard species correlation networks (SCN) [51, 56]. In the present study, it is used to construct OMU co-occurrence (correlation) networks. I realize there are critics on the usage of Spearman’s coefficients, which are particularly appropriate when the sparsity of data is high (lots of low abundance species) [57]. Indeed, to build CPN/HSN, Spearman’s coefficient, actually, even the species correlation network, are not required, and alternative methods for building the networks may be used. The reason I still use Spearman’s correlation coefficients is that the functional gene clusters (MFGC, MF, MP) do not suffer from the limitations of the species abundance data used for building species correlation or OTU correlation networks. In fact, in type-I MFGC, the gene abundance is not used at all, and therefore, it is not compositional data. Furthermore, sparsity with the MFGC/MF/MP datasets is very low, and the numbers of MFGC/MF/MP are rather close among samples. To keep the consistent treatments for both type-I MFGC and type-II MFGC data, I decided to use Spearman’s coefficients in this study.

The computational code for calculating Spearman’s correlation coefficients and building standard correlation networks are available in multiple public domains (e.g., https://igraph.org/) [51]. From the SCNs, the previously, briefly described algorithms for building core/periphery and skeleton networks are applied to reconstruct the corresponding CPN and HSN networks [16, 20], and computational procedures and codes can be found in Ma and Ellison [16]. The computational codes for detecting special trio motifs and PN ratios were provided in Ma and Ye [51]. The program I used to fit Sloan model was published in Burns et al.[45]. Finally, the source code for performing stochasticity analysis with the NSR was from Ning et al. [19].

Randomization tests with 1000 times of re-sampling are performed to determine the change of shared core/periphery nodes between the healthy and diseased treatments, two algorithms (A1 = remix of MFGCs; A2 = remix of samples) developed by Ma et al. [58] are used to implement the randomization tests. A significant reduction of shared core or periphery between the healthy and diseased samples indicates statistically significant effects of diseases (obesity, type-2 diabetes, and IBD) on the core/periphery structures of MFGC/MF/MP and may have significant biomedical implications. Furthermore, I adapted standard randomization tests for testing the difference of CPN/HSN properties between the healthy and diseased treatments. 1000 times of re-sampling were used to reconstruct 1000 CPN/HSN, and their properties were computed and compared (*see* Ma 2020b for detailed computational procedures). Throughout the study, most significance tests for the differences between treatments were performed with the randomization (permutation) tests [20, 58, 59], which possess two advantages: One is that the method can accommodate the possible differences in the sample sizes between treatments. Another is that statistically significant difference is reported when the inter-treatment difference exceeds the intra-treatment variation as exhibited by pseudo *P*-value. Therefore, in the case of this study, the pair-wise comparisons between the healthy and diseased treatments in terms of various network properties should reflect the disease effects, or more accurately effects associated with diseases. Nevertheless, since currently, the causal relationships in most microbiome-associated diseases such as IBD and diabetes are unclear, the differences revealed by the randomization tests only signal the differences associated with diseases (even though the term of disease effects may be used).

## Results

### The neutral-theoretic analysis of human gut metagenomes

#### Classifications of neutral, below-neutral and above-neutral OMUs (MGs/MFGCs)

We investigated the neutrality of human gut metagenomes with Sloan [42, 43] near neutral model at two levels, the MG (metagenomic gene) and Type-II or abundance-based MFGC (metagenome functional gene cluster). As explained previously, Type-I or non-abundance MFGC and MF/MP cannot be approached with Sloan neutral model because Type-I or non-abundance MFGC/MF/MP ignored the gene abundances.

**Fig. 2:**
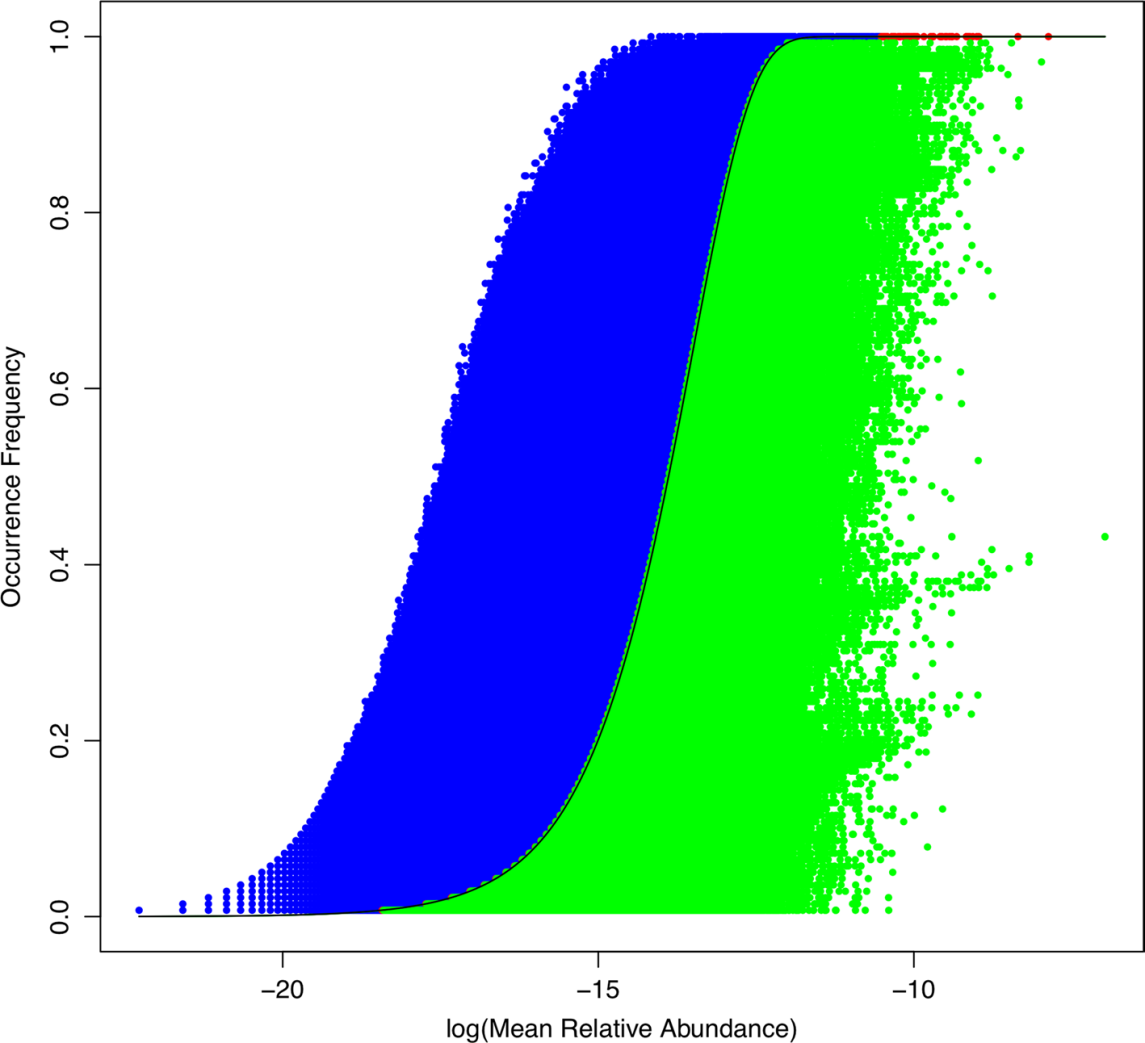
Sloan near-neutral model fitted to the MGs (metagenomic genes) in human stool metagenomes (the 1st dataset in Table 1) showing the neutral MG (few and negligible red dots), above-neutral (blue dots at the left side) and below-neutral (green dots at the right side)

**Fig. 3:**
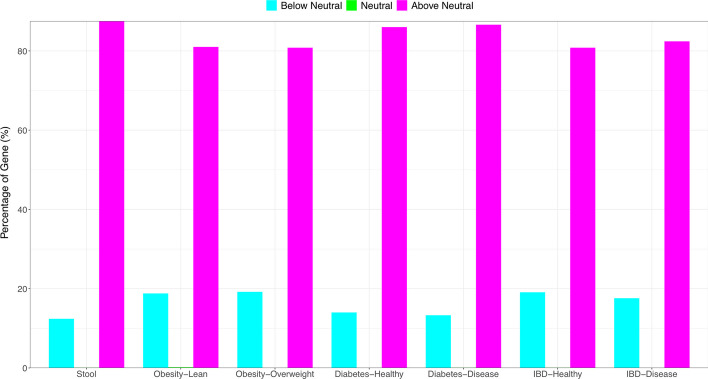
Percentages of the three categories of MGs (metagenomic genes) classified by Sloan near neutral models: below-neutral (cyan blue bar), neutral (green bar showing neutral MGs), and above-neutral (magenta bar) (drawn based on Additional file 1: Table S1). Note that the green bars for the neutral percentages were too low to be visible here

At MG level (Additional file 1: Table S1), the average percentages of neutral, under-neutral (negatively selected) and above-neutral MGs across 7 metagenome datasets is less than 0.1% (5024 out of 5,864,966), 16.3% and 83.6% respectively. This result suggests that majority of MGs are above-neutral. Figure 2 illustrated the Sloan model graph fitted to the MGs of the independent stool treatment, which was based on the metagenomes of 139 healthy individuals (see Table 1). Figure 3 illustrated percentages of the three categories of MGs for each of the seven metagenome treatments (datasets).

**Fig. 4:**
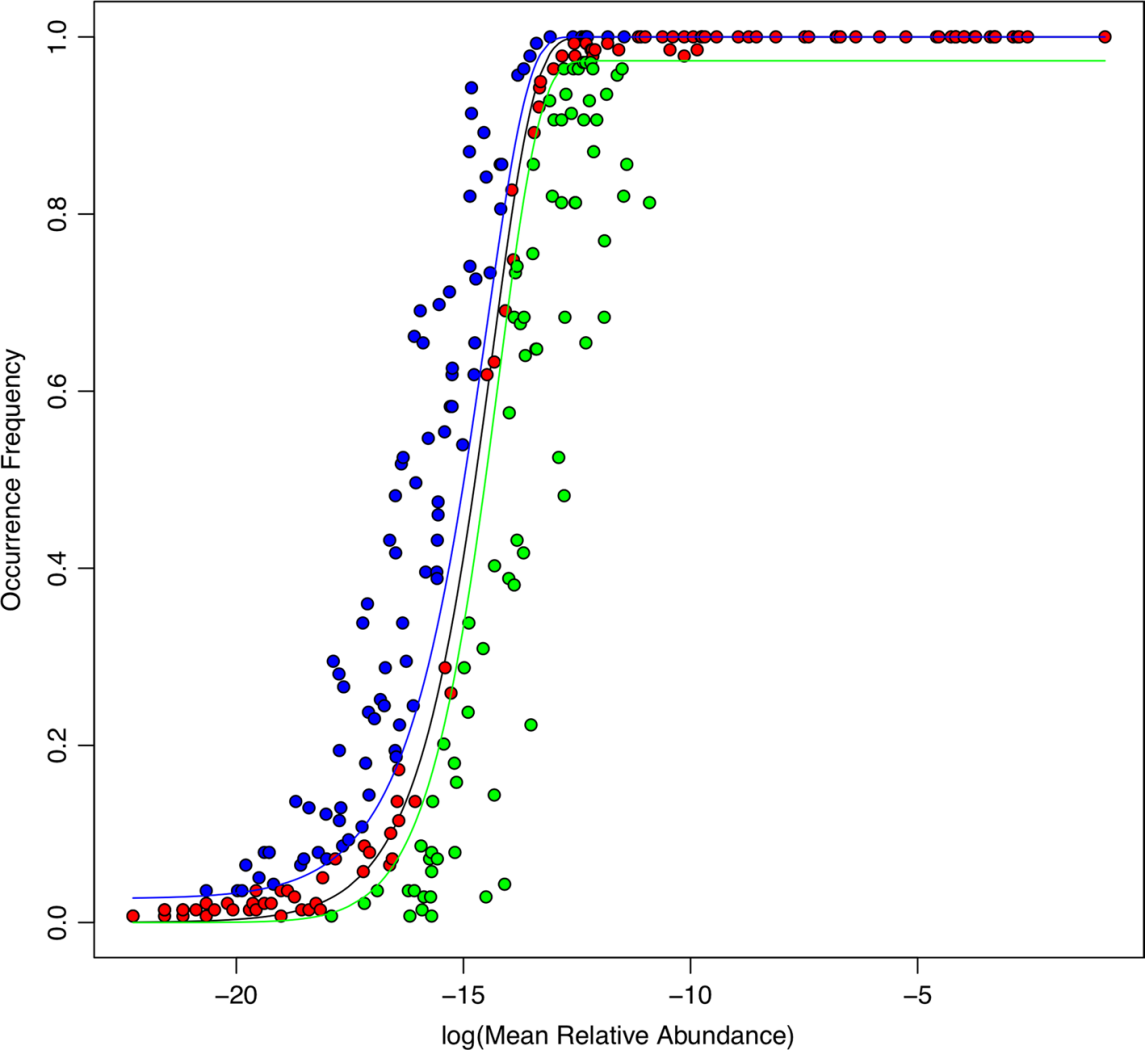
Sloan near-neutral model fitted to the MFGCs (metagenome functional gene clusters) of the human stool metagenome samples (the first dataset in Table 1) (based on KEGG database) showing the neutral MFGC (red dots), above-neutral (left side blue dots) and below-neutral (right side green dots)

At MFGC level (Additional file 1: Tables S2), the average percentages of neutral, below-neutral and above-neutral MFGC across seven metagenome datasets in terms of the metagenomic functions (eggNOG database) were 62.4% (212 out of 341), 12.2% and 25.4% respectively. In terms of the metabolic pathways (KEGG database), the average percentages of neutral, under-neutral and above-neutral MFGC were 49.4% (135 out of 274), 20.6% and 30.1% respectively. This indicates that neutrality at the functional gene cluster level (i.e., MFGC) is significantly raised, compared with that at the metagenomic gene (MG) level, and the level of increase for metagenomic function (eggNOG) is about 10% more than for the increase for metabolic pathways (KEGG), exceeding approximately 50% in both the cases. Figure 4 illustrated the Sloan model graph fitted to the MFGCs of the independent stool treatment, in which the neutral MFGCs reached 57% or 192 MFGCs (Additional file 1: Table S2).

One interpretation for the above observations is that, as the clusters of same or similar functional genes (pathways), there are enormous redundancies within MFGC. The redundancy can be considered as some kind of equivalency among metagenomic genes. It is the huge redundancy or equivalency that leads to the significant rising of neutrality from less than 0.1% at the MG level to near (KEGG) or more than (eggNOG) 50% at the MFGC level. One may argue that the high neutrality at MFGC is an artifact due to data aggregation effects since MGFC were clustered according to similarity in functions of MGs. Still, the analysis suggests that there are huge redundancies both within MFGC and also among MFGCs.

**Fig. 5:**
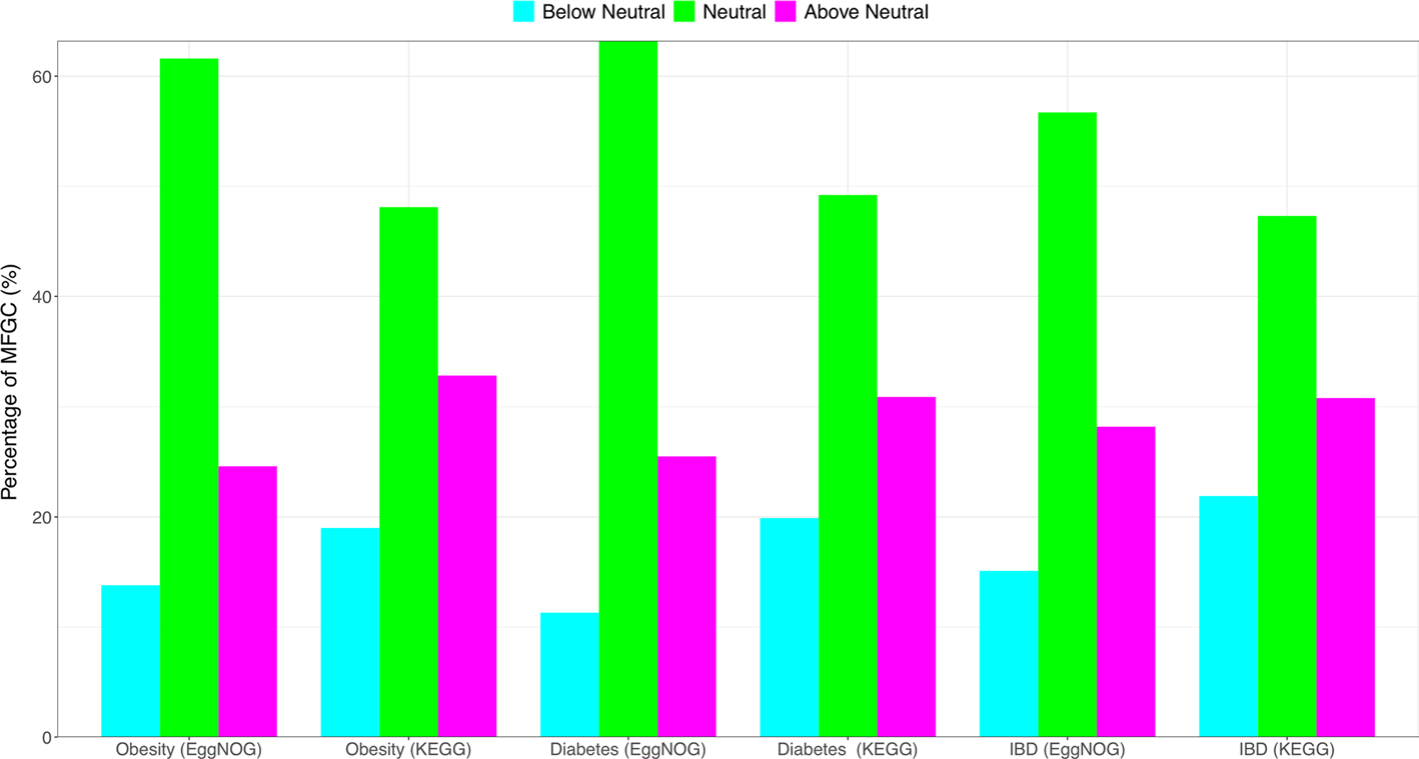
Percentages of three categories of MFGCs (metagenome functional gene clusters, based on KEGG and EggNOG database) classified by Sloan near neutral models: below-neutral (cyan blue bar), neutral (green bar showing neutral MGFCs), and above-neutral (magenta bar)

I further built Sloan neutral model for each pair of the healthy versus diseased treatments (obesity, type-2 diabetes, and IBD), with the healthy as source community and diseased metagenome as destination community (Additional file 1: Table S3). The results are very similar to the previously interpreted results separately built for each of the 7 treatments (metagenome datasets). The differences in percentages of neutral, below neutral and above neutral were rather small (within 3%). Figure 5 illustrated percentages of the three categories of MFGCs for the three diseases (obesity, type-2 diabetes, and IBD), in terms of metagenomic function (eggNOG database) or metabolic pathway (KEGG).

Additional file 1: Table S4, a batch of Excel sheets in the OSI (online supplementary information), listed the neutrality status (neutral, below, and above) of each MFGC in each treatment, in terms of either metagenomic function or pathway. The terms of above-neutral and below-neutral are borrowed from the terminology for characterizing selection versus neutral drift in population genetics [5].

In summary, I demonstrated the applicability of Sloan’s near-neutral model to seven OMU treatments just as its applicability to the OTUs. Sloan models classified all MGs/MFGCs as neutral, below-neutral and above-neutral: the neutral MGs are negligible but neutral MFGCs are near 50% or more, suggesting enormous functional gene redundancy.

#### Randomization tests of Sloan (2007) near-neutral model parameters

I performed standard randomization tests (with 1000 times of re-sampling) for the effects of diseases (obesity, type-2 diabetes, and IBD) on the neutrality, specifically Sloan neutral model parameters. Additional file 1: Table S5 listed the test results for the differences between the healthy control and diseased treatment within each of the three sets of datasets, and only one parameter (below-neutral MFGC number) exhibited significant difference in the case if IBD. Since I only have three datasets, I do not draw any conclusion on the generality of disease effects on neutrality. Instead, the objective of this study is focused on demonstrating useful methods.

### Critical network structures (Trios/PN-Ratio/CPN/HSN) in MFGC co-occurrence networks

#### Special trio motifs and PN ratio

As mentioned in previous introduction, the huge number of MGs makes it hardly possible to build complex networks on common computational platforms. For example, in the case of this study, the disk space needed to store Spearman’s correlation coefficients I computed for one MG dataset exceeded 4 Terabytes, which made it impossible to complete consequent network analysis. To deal with the computational difficulties, I build networks with MFGC and MF/MP in this study. As argued previously, the loss of insights from using MFGCs rather than MGs is minimum.

Additional file 1: Table S8A (“trios without MAO handle”—the most abundant MFGC is a node of trio) and S8C (with MAO handle—the most abundant MFGC is linked to trio via a separate handle or link) displayed the numbers of 15 kinds of special trio motifs. Additional file 1: Table S8B (without MAO handle) and Additional file 1: Table S8D (with MAO handle) displayed the randomization tests results for those trios, testing whether there were significant differences in the trio numbers between the healthy and diseased treatments. These special trio motifs, as fundamental building blocks for all other network modules, may act as in silico biomarker for signaling disease effects. Although there was not a consistent trio motif that is significantly different between the healthy and diseased treatments across all datasets, on average, there was at least one kind of trio motif that made the difference for each disease case, suggesting the potential of finding an effective in silico biomarker with the trio-motif detection technique.

Additional file 1: Table S9A showed the PN (positive to negative) ratio for each of the seven treatments and Additional file 1: Table S9B showed the results of randomization tests for the differences between the healthy and diseased treatments in each of the three diseases. While obesity case did not exhibit significantly different PN ratio between the lean and overweight treatments, the PN ratios were significantly different between the healthy control and diseased treatments in the cases of type-2 diabetes and IBD.

#### Core/periphery network (CPN) properties and shared core/periphery nodes

Additional file 2: Table S10 (Excel file) listed the categorization of all network nodes (MFGCs) as either core or periphery nodes for each of the seven treatments. The information can be useful for understanding the biomedical insights of MFGCs. Additional file 1: Table S11A exhibited the core/periphery network (CPN) properties for each of the seven treatments, including core strength (ρ), ratio of C/P [core/(core + periphery)], density matrix (internal strength), PN ratio for the core/periphery structures, nestedness, etc. Additional file 1: Table S11B exhibited the *p*-values from testing the differences in the CPN properties between the healthy and diseased treatments. In most cases, it appears that the CPN properties did not exhibit significant differences between the healthy and diseased treatments.

Besides testing the differences in the CPN properties between the healthy and diseased treatments as interpreted above, I also performed shared core/periphery nodes analyses between the healthy and diseased treatments based on an approached developed in Ma et al. [20] and further tested in Ma [58]. The shared core/periphery analysis answers the question: whether or not the similarity between the healthy and diseased treatment decreased or increased more than the change by pure chance, in terms of either core, periphery, or their total (core + periphery). As demonstrated in Additional file 1: Table S12, the most significant declines of shared core/periphery nodes between the healthy and diseased treatments occurred in Type-II or abundance-based MFGC, especially in eggNOG-indexed metagenomic functions. The differences were less significant in Type-I or non-abundance MFGC, which should be expected due to its ignorance of the gene abundances of MGs within each MFGC (only the number of MG kinds were considered in non-abundance MFGC). These results indicated the potential of using the shared core/periphery analysis as a tool for predicting disease risks.

#### High-salience skeleton networks

Additional file 1: Table S13A exhibited the high-salience network (HSN) properties for each of the seven treatments, including percentages of high-salience links (out of total links), statistics of salience value (such as mean, maximum, skewness, kurtosis), and network assortativity (measure of network resilience). The randomization tests for the differences in these network properties showed similar patterns as in previous CPN properties (Additional file 1: Table S13B).

**Fig. 6:**
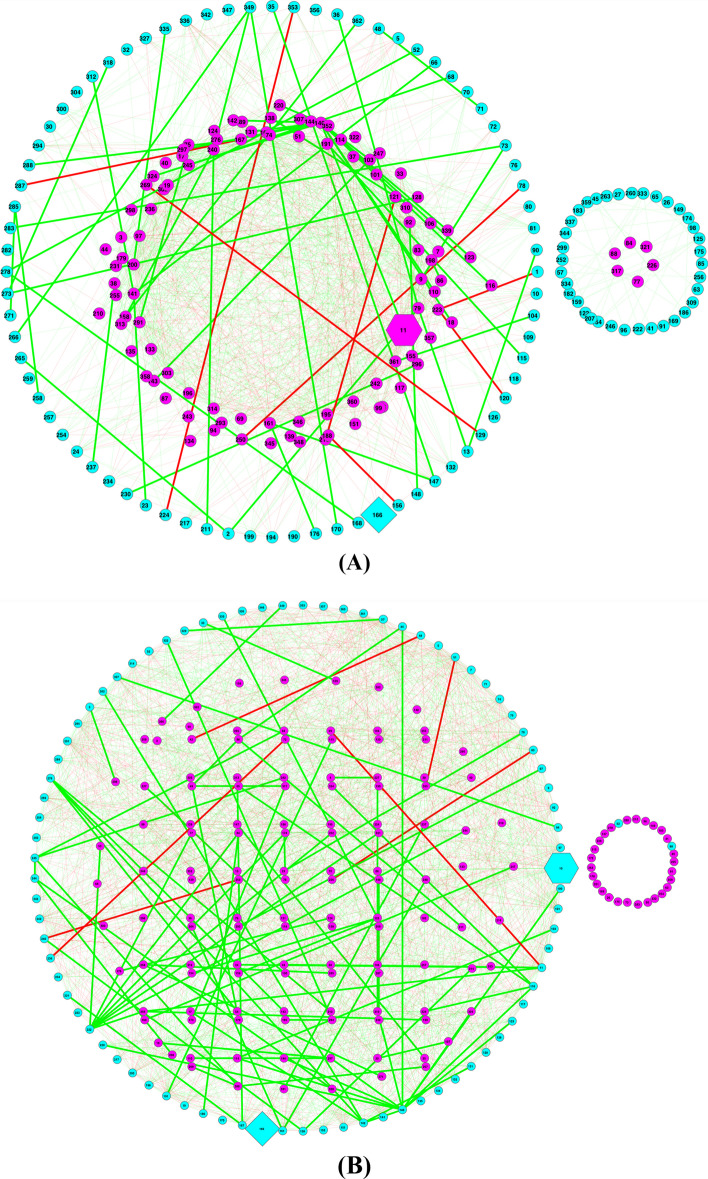

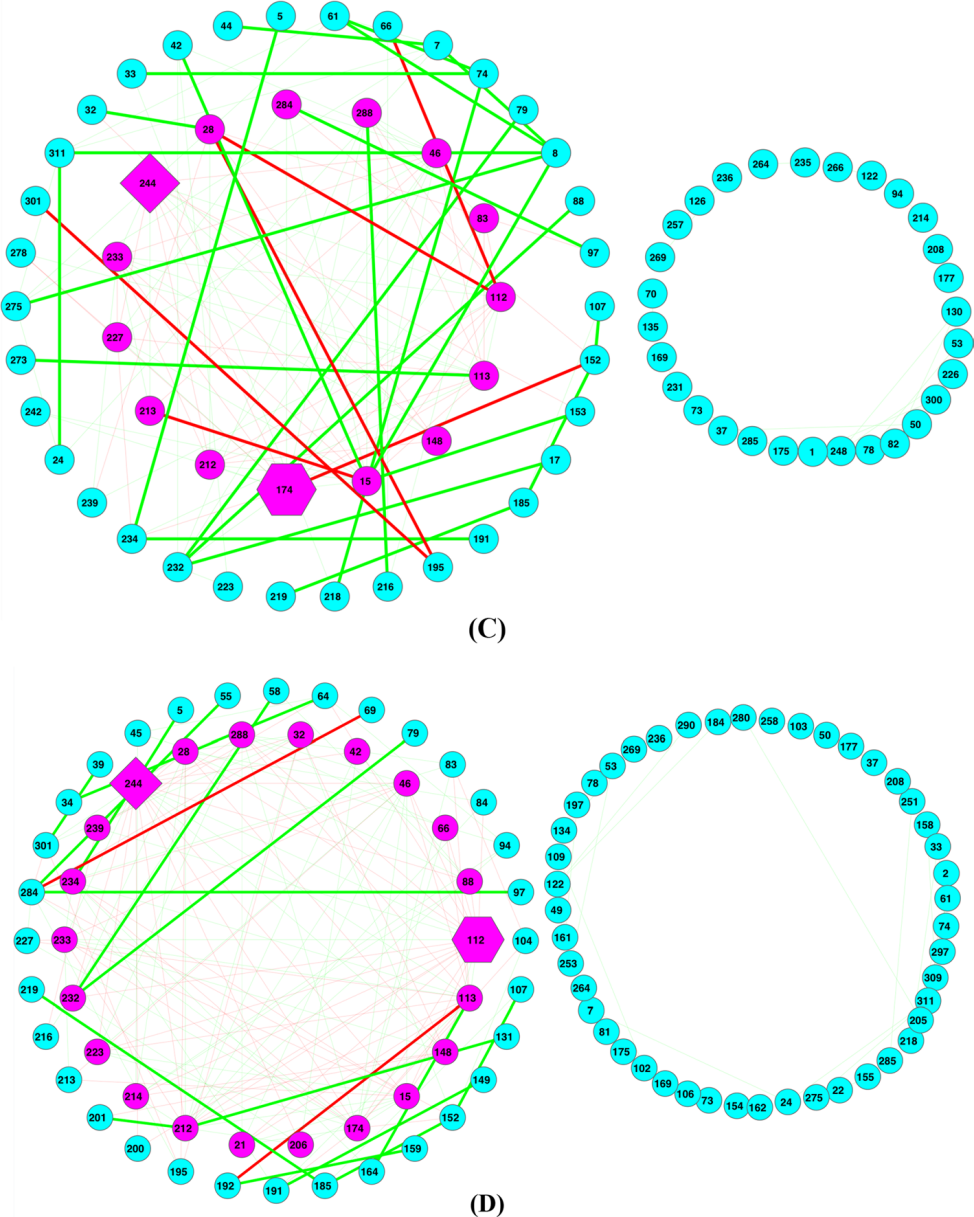
The MFGC-II (Type-II MFGC or abundance-based MFGC) networks for the lean and overweight treatments of the obesity dataset (**A-D**); Legends: *core* nodes in magenta (located mostly in center), *periphery* nodes in cyan, green links for positive correlations and red links for negative correlations, thicken links are high-salience skeletons (backbones), hexagon-shaped node for network *hub*, diamond-shaped node for the most abundant MFGC in the network, cycle for regular nodes (either core in magenta or periphery in cyan). (**A**) MFGC-II (abundance-based MFGC) network based on functional eggNOG database—Lean treatment of Obesity dataset (**B**) MFGC-II (abundance based MFGC) network based on eggNOG database—Overweight treatment of Obesity dataset; (**C**) MFGC-II (abundance-based MFGC) network based on KEGG database—Lean treatment of Obesity dataset; (**D**) MFGC-II (abundance based MFGC) network based on KEGG database—Overweight treatment of Obesity dataset

Figure 6 shows the Type-II or abundance-based MFGC network graphs (Fig. 6A, B based on eggNOG functional database; Fig. 6C, D based on KEGG pathway database) for the obesity dataset (lean vs. overweight), displaying various components such as core/periphery nodes, backbones, and hub.

In summary, the CPN and HSN offer tools to assess the *selection* effects [“anti-equivalence” including node heterogeneity and link (interaction) asymmetry] in MFGC networks from node and link perspective, respectively. Integrated with neutral modeling, the CPN and HSN cover the full spectrum of the four processes underlying the patterns of metagenome assemblages. The NSR approach, to explain below, further cross-verifies the results from neutral-theoretic and network analyses by presenting an independent cross-verification of the relative strength between stochasticity and deterministic selection. Moreover, the trio-motif and PN ratio, explained previously offer potential in silico biomarkers for disease-diagnosis and risk prediction.

### Critical network structures (Trios/PN-ratio/CPN/HSN) of MF/MP co-occurrence networks

The MFs (metagenome functions) and MPs (metagenome pathways) are building blocks (set elements) of MFGCs, which are sets (clusters) of MFs or MPs (see Fig. 1). Additional file 1: Table S14 displayed the descriptions of 24 MFs (e.g., nucleotide transport and metabolism) and 37 MPs (e.g., genetic information processing). Additional file 1: Table S15A & Table S15C exhibited the numbers of 15 special trios, respectively. Additional file 1: Table S15B and Table S15D exhibited the results (*p*-values) of randomization tests for the differences in the numbers of various trios between the healthy and diseased treatments. Additional file 1: Table S16A and Table S16B listed the PN ratios in the MF/MP networks as well as the *p*-values of the randomization tests for the differences in the PN ratios between the healthy and diseased treatments. Compared with the corresponding results of MFGCs in the previous section, the differences in special trios and PN ratio in the MF/MP networks are less significant, which should be expected because MFs/MPs are at the foundational level (set elements of MFGCs) and should be more robust (stable or less variable) than MFGCs.

Additional file 2: Table S17 (Excel file) classified the MF/MP as core or periphery nodes respectively. The core status of MFs/MPs suggests the densely connected (inter-dependent) functions/pathways, while periphery status suggests loosely connected or even independent MFs/MPs functions/pathways. Therefore, the core/periphery statuses of MFs/MPs should be of potentially important biomedical significances.

Additional file 1: Table S18A and Table S18B exhibited the results of CPN properties of MF/MP networks. Additional file 1: Table S19 listed the shared core/periphery analysis between the healthy and diseased treatments, using the same algorithms for the previous shared core/periphery analyses for the MFGC networks. It turned out that in the MF/MP networks, shared core/periphery nodes declined less than by chance. In other words, the shared MF/MPs between the healthy and diseased treatments are near constant, which could be interpreted by the foundational nature of MFs/MPs as explained previously—the foundation should be more robust against perturbations such as diseases.

Additional file 1: Table S20A and Table S20B exhibited the results of HSN properties of MF/MP networks. In general, the proportions of the CPN/HSN properties of MF/MP networks with significant differences between the healthy and diseased treatments are similar with those of the MFGC networks.

**Fig. 7:**
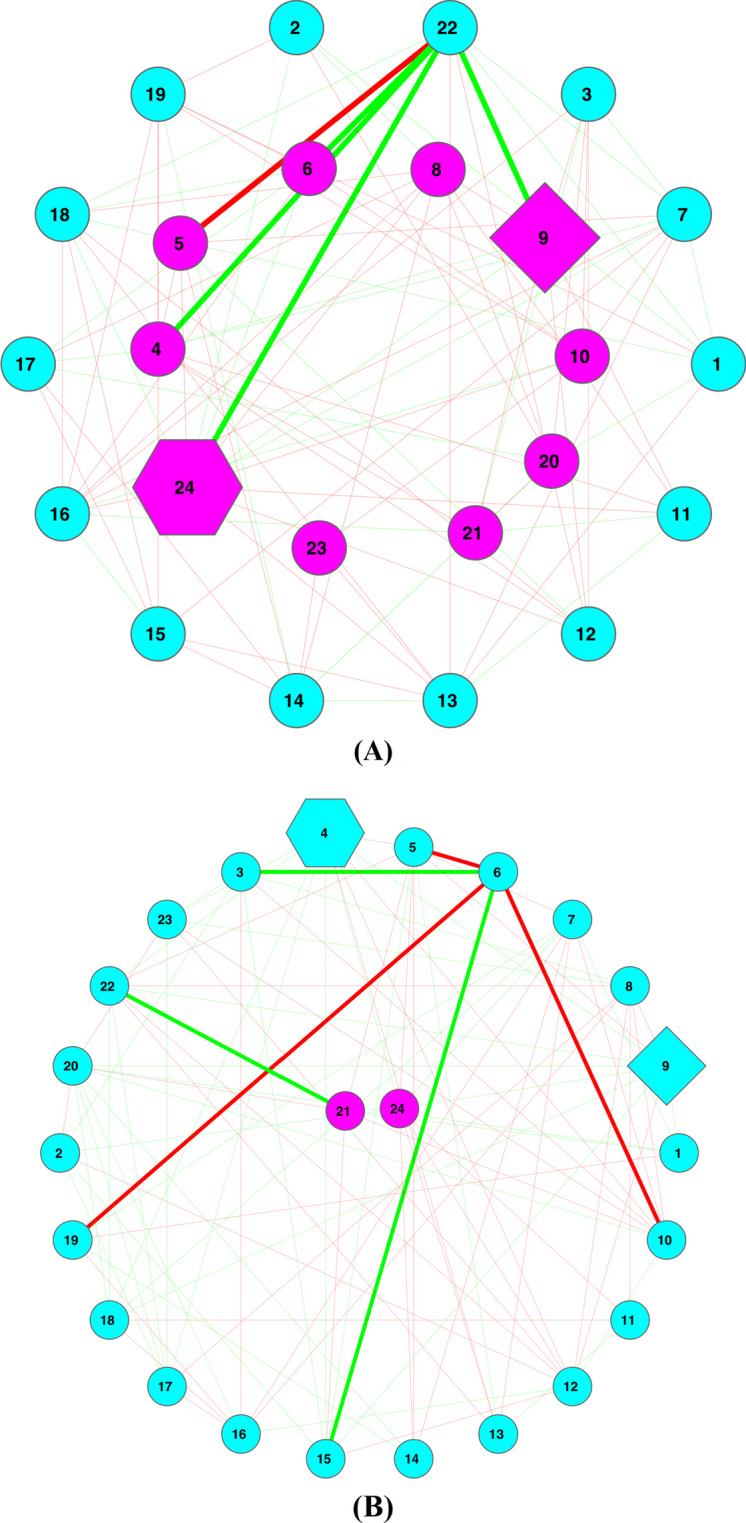

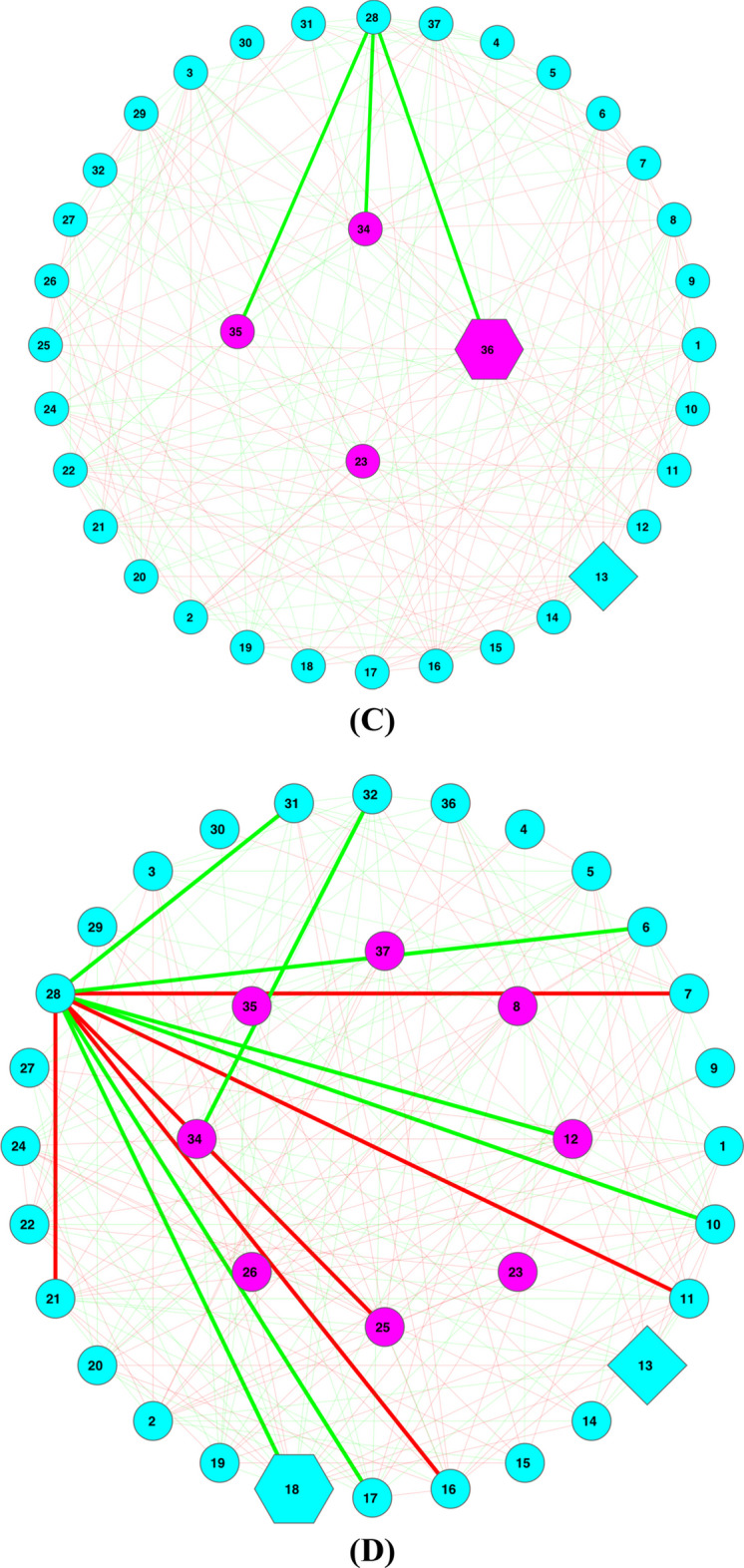
The MF-I (Type-I metagenomic function or non-abundance based, aligned with eggNOG database) and MP-I (type-I metagenomic path, based on KEGG databases) networks for the lean and overweight treatments of the obesity dataset (**A**-**D**); Legends: *core* nodes in magenta (located mostly in center), *periphery* nodes in cyan, green links for positive correlations and red links for negative correlations, thicken links are high-salience skeletons (backbones), hexagon-shaped node for network *hub*, diamond-shaped node for the most abundant MF/MP in the network, cycle for regular nodes (either core in magenta or periphery in cyan). (**A**) MF-I (non-abundance based MF) network based on eggNOG database—Lean treatment of Obesity dataset; (**B**) MF-I (non-abundance based MF) network based on eggNOG database)—Overweight treatment of Obesity dataset; (**C**). MP-I (non-abundance based MF) network based on KEGG database—Lean treatment of Obesity dataset; (**D**) MP-I (non-abundance based MF) network based on KEGG database—Overweight treatment of Obesity dataset

In summary, for MF/MP networks, the CPN and HSN properties exhibited similar patterns with MFGC networks in terms of the level of differences between the healthy and diseased treatments. However, in the case of special trio motifs, PN ratio and shared core/periphery nodes the MF/MP networks exhibited more robust patterns than the MFGC networks due to their foundational nature (also see Fig. 1). Figure 7 shows the MF (Fig. 7A, B, based on eggNOG) and MP (Fig. 7C, D, based on KEGG) network graphs for the obesity dataset (lean vs. overweight), displaying various features such as core/periphery nodes, backbones, and hub.

### Integration of neutral-theoretic modeling and core/periphery networks

In previous sections, although the Sloan neutral model and CPN/HSN were applied independently, the results from both approaches complement each other *indirectly*, with neutral modeling covering the three of four processes (drift, mutation and dispersal) and CPN/HSN covering the remaining selection process. I further performed direct integration of both the approaches by applying Sloan model to the core and periphery of MFGC networks respectively (Additional file 1: Table S6). Randomization tests were conducted to test the difference between the healthy and diseased treatments in the distribution of neutral versus non-neutral core/periphery MFGCs (Additional file 1: Table S7). For example, in the case of type-2 diabetes, in both core and periphery, there were more positivity selected MFGCs in the healthy treatment.

### Stochasticity analysis of the human metagenomes with the NSR

While the previously interpreted results from Sloan neutral model and CPN/HSN have painted a general picture on the relative balance between stochastic neutrality versus deterministic selection, the final sub-section of the results section is aimed to cross-verify the results from the previous sub-sections. I apply Ning et al*.* [19] framework for stochasticity analysis to gauge the relative strength of stochasticity (stochastic neutrality), known as *normalize stochasticity ratio* (NSR), which ranges between [0, 1] with zero representing for the complete deterministic selection (lack of stochasticity) and one for complete stochasticity.

As shown in Additional file 1: Table S21, the values of NSR for most treatments were rather high for the MFGC, exceeding *0.5* in all treatments and ranged from 0.701 to 0.947 suggesting that stochastic neutrality surpasses the deterministic selection. The pattern is consistent across all three schemes with intra-healthy treatment, intra-diseased treatment and inter-healthy and diseased treatments, and the differences among the three schemes are minor. Compared with the percentages (49.4–62.4%) of neutral MFGCs from previous Sloan models, the stochasticity level revealed by NSR (70.1–94.7%) is slightly high, but the trends of patterns revealed by both approaches are consistent.

For the MG, the overall values of NSR ranged from 0.169 to 0.213, which is significantly smaller than *0.5* (the level of equality between neutrality and selection). According to previous results from Sloan model, the sum of the percentages of neutral MGs and below-neutral MGs was 16.4% and the percentage of above-neutral MGs was 83.6%. Both the results are again consistent, supporting the exceptionally high deterministic selection and low stochastic neutrality at MG level. In fact, at the MG level, the stochasticity levels from Sloan model and NSR are indeed rather close, i.e., 16.4% (0.164) versus [0.169, 0.213].

Additional file 1: Table S22 listed the *p*-values from comparing the differences in NSR between the healthy and diseased treatments, and the percentages with significant differences ranged from 60 to 100%, suggesting significant differences in disease effects in general, although the percentages varied from disease to disease.

In summary, the stochasticity analysis with NSR here is aimed to cross-verify the results from previous sections based on Sloan neutral modeling and CPN/HSN networking analysis. The cross-verification is important because, although both Sloan model and CPN/HSN separately offered solid and quantitative characterizations in their own domains, their integrated applications for assessing the full spectrum of the four-process synthesis, i.e., the balance between stochastic drifts versus deterministic selection is imperfect. Strictly speaking, there may be overlap between the measurements (percentages of neutral, below-neutral and above-neutral MUs) form Sloan near neutral model and the metrics (e.g., high-salience backbones, core/periphery structures) from CPN/HSN. In the case of Sloan model, it is possible to distinguish the neutral versus selection (above-neutral MUs). Similar distinction is not possible with CPN/HSN approaches to deterministic selection. The NSR approach demonstrated here offer an alternative tool to evaluate the relative strength between stochasticity and selection.

## Discussion

The present study is aimed to develop and demonstrate a set of ecological and network approaches to identifying critical ecological processes (mechanisms) and network structures underlying the patterns of metagenomes represented with various levels of OMUs (specifically, MG/MFGC/MF/MP). These approaches have proven to be effective for analyzing the 16s-rRNA sequencing reads from amplicon sequencing technology, particularly for testing the effects of microbiome associated diseases [16, 20, 31–40, 50, 58, 60, 61]. Therefore, if these approaches are found applicable for the metagenomes from whole-genome (shotgun sequencing) technology, we are one step closer to develop a unified microbiome ecology of microbial OMUs (units of metagenomic genes) and OTUs (units or taxa of microorganisms). I demonstrated the feasibility of the unified approaches with seven datasets of the human gut metagenomes including three diseases (obesity, type-2 diabetes, and IBD) and an independent stool metagenome dataset. Although I could not draw precise conclusions on their effectiveness in detecting disease effects as previously demonstrated with 16s-rRNA datasets due to the limited number of datasets analyzed in this study [20], the general applicability of the approaches in analyzing the critical ecological mechanisms and network structures for both microbial gene and taxa data has been established firmly through this study.

Specifically, Sloan [42, 43] near neutral model can be utilized to classify all MGs/MFGCs as neutral, under neutral or above neutral. This information, in our opinion, is of important ecological or biomedical significance since the neutral or non-neutral status indicates the relative ‘position’ of each MG (MFGC) in the balance between stochastic neutral drifts versus deterministic selection forces. Moreover, it was discovered that at the MG level, positive selections were dominant and neutral drifts were negligible; however, at the MFGC level, the neutral drifts reaches approximately a half or more in terms of the proportions of neutral MFGCs. I postulated it is the enormous metagenomic gene redundancies within MFGC and possibly among MFGCs that lead to the rising of neutrality. As a side note, due to enormous number of MGs, Sloan neutral model was the only computationally feasible neutral model for the metagenome at the MG level, which was also the primary reason for our choice of Sloan model.

While Sloan model was intended to assess stochastic *drifts* directly and *selection* effects indirectly (assuming that both drifts and selections are additive exclusively, which may not be totally correct), the trio motif detection, CPN, and HSN were designed to detect node heterogeneities and asymmetric interactions (i.e., selection effects). For example, the distinctions of core/periphery nodes revealed the nestedness and heterogeneities in the CPN networks of MFGC (MF/MP) from node perspective, which are closely related to network stability (or stability of metagenomes) and may have important health implications. The high-salience skeletons identified from the HSN analysis indicate the critical interactions (connections) in the MFGC (MF or MP) networks from link perspective, similar to the critical role of backbones in transportation networks. Integrated together, the heterogeneity and/or asymmetry in both nodes and links can be considered as the signatures of the selection in metagenomes. The trio motifs (as the fundamental building blocks for more sophisticated network modules) may act as potential in silico biomarker for detecting disease effects, and the PN ratio may signal the balance of cooperative versus competitive interactions in MFGC/MF/MP networks.

I subscribe to Vellend [6, 7] and Hanson et al. [8] synthesis for community ecology and microbial biogeography, actually, I translate it into the synthesis for “*assemblage of metagenomes*” that can be considered as the counterpart of metacommunity in community ecology. I postulate that the similar four processes (including drifts, selection, dispersal and mutation) in the assemblage of metagenomes that shape the spatial distribution patterns (inter-subject distribution) of human metagenomes and possibly their dynamics. The previously described and demonstrated approaches offer a set of tools to assess and interpret the relative importance of the four processes and consequently shed lights on the mechanisms underlying the patterns of metagenomes. Nevertheless, I caution that the four-process synthesis is largely qualitative and quantitative characterizing of the processes is rather challenging.

A primary objective of this study is to enrich the repertoire for performing post-bioinformatics metagenome analyses, especially the ecological and network analyses. In existing literatures of microbiome research, the ecological and network analyses for the OMUs, compared with those for the OTUs from amplicon sequencing technologies, have been relatively fewer. In my opinion, at least, there are two possible reasons for this lag in the field of metagenome research. One may be the concern that metagenomic genes are not organisms, and therefore, the applicability of ecological approaches could be in question. This study demonstrates the feasibility for developing a unifying microbiome ecology covering both microbes and the metagenomes they carry. Another challenge is the huge numbers of MGs, which are in tens of millions and make it hardly possible to apply network analysis techniques directly. The definitions of MFGC/MF/MP circumvented the obstacle as demonstrated in this study. As argued previously, the reality that for most metagenomic genes, the only information known is a label (gene number) and the MFGCs they belong to, plus the enormous functional redundancy, the loss of insights from using MFGCs (or MFs/MPs) as proxy of MGs should be tolerable.

A limitation of this study is the limited number of the metagenome datasets (only 3 sets of 7 treatments), even though their qualities are among the best in existing literature. The limited datasets makes it hardly possible to draw general conclusions on the effects of microbiome-associated diseased on the human gut metagenomes. For this limitation, the objective of this study was set to demonstrate the feasibility of the proposed approaches. Nevertheless, the general applicability of the proposed approaches should have been firmly verified in previous sections.

Another limitation of this study is the lack of explicit modeling of evolution, although our article does deal with the assessment of selection. Our assessment of selection based on the metagenome abundance data is therefore *indirect* since both Sloan [42, 43] near-neutral model and Ning et al. [19] NSR framework measure the relative balance between deterministic selection and stochastic drifts, which are the consequences or effects of selection/drifts, rather than selection/drifts per se. This lack of direct modeling of evolution also made our work somewhat “detached” from some prevalent theories, most notably, the holobiont/hologenome (host and their symbionts/the total genomes), ITSNTS (it’s the song not the singer)—it’s the function of microbiota versus not the taxa composition that is highly redundant, community genetics/evolution [62–66]. I hope that future studies will fill the gap between our approaches and those important theories. In our opinion, animal microbiomes, especially the microbiome of wildlife, should be better systems than the human microbiomes for exploring the evolution of microbiomes [65]. Indeed, the widely used intervention measures such as surgery, antibiotics, and now fecal transplantation introduce engineering style renovation, which can be rather different from naturally occurring evolution, not to mention the potential influences of emerging new technologies such as gene editing and even AI (artificial intelligence) in future.

Still another limitation of this article is the terminology I suggested lack rigorous definition, not to mention precise quantification, especially the concept of OMU. As mentioned previously, the OMU and OTU are not perfectly reciprocal. For example, the calculation of OTUs usually has unified or consistent algorithms (procedures) to delineate various levels of OTUs, but the OMUs we defined in this article do not have a unified algorithm to identify and catalogue. Since the objective of this article is to demonstrate the applicability of complex networks analysis and neutral-theoretical analyses, the introduction of the new terminology is aimed to facilitate the analyses, especially the presentation of the models and results, which are traditionally described with OTUs in the existing literature. Without using the concept of OMU, one may need to repeatedly explain same computational procedure for each of the metagenomic unit, starting from MG, through MFGC, to MF/MP.

## Conclusions

The repertoire for post-bioinformatics analyses of metagenome datasets from whole-genome sequencing technology is much smaller than those for amplicon sequencing (e.g., 16s-rRNA) for two main reasons. The first is the uncertainty around the applicability of ecological theories given that metagenomes are assemblages of genes, rather than of organisms. Second, orders of magnitude more MGs (metagenomic genes) than the numbers of microbial species make the analyses, especially ecological network analysis, extremely challenging computationally. I demonstrated the applicability of a set of ecological/network approaches (including Sloan near-neutral model, core/periphery network (CPN), high-salience skeleton network (HSN), trio-motif and PN ratio) for assessing and interpreting the relative importance of the four processes (drift, selection, mutation, and dispersal) in shaping the patterns of “assemblage of metagenomes” (i.e., the counterpart of ecological *metacommunity*). Technically, the introduction of metagenome functional gene clusters (MFGC) as proxy of MGs readily circumvents the computational challenge.

In conclusions, the ecological concepts, models and theories including diversity, heterogeneity, stochasticity (Sloan near-neutral model, Ning et al. stochasticity framework), complex networks (core/periphery network, high-salience skeleton network, special trio-motif, PN ratio, etc.) are demonstrated to be applicable to whole-genome metagenomic sequencing data or the OMU datasets, just as they are proved to be suitable for analyzing OTU datasets. The introduction of OMU or MU (as its shorthand) concept does facilitate the applications. Therefore, the goal as stated in the title of this article, unifying the medical ecology of metagenome (OMUs) and microorganisms (OTUs), and of microbiome (microbes) and macrobiomes (macrobes) seems to be both feasible and meaningful.

### Supplementary Information

**Additional file 1.** Table S1–S22.

**Additional file 2.** Supplementary Tables.

## Data Availability

All the datasets analyzed are publicly available (see Table 1 for the sources). Virtually all codes have been released in our previous publications, as documented in the section of datasets and methods.

## Abbreviations

CAG: Co-abundant gene group
CPN: Core/periphery network
eggNOG: Evolutionary genealogy of genes nonsupervised orthologous groups
HSN: High-salience skeleton network
KEGG: Kyoto Encyclopedia of Genes and Genomes
MAO: Most abundant OTU
MG: Metagenomic gene
MGA: Metagenomic gene abundance
MF: Metagenome function
MP: Metagenome pathway
MFGC: Metagenome functional gene cluster
MGS: Metagenomic species
OMU: Operational metagenomic unit
MU: Metagenomic unit
OTU: Operational taxonomic unit
SCN: Species correlation networks
UNTB: Unified neutral theory of biodiversity
