# Supplementary material for: Towards a unified medical microbiome ecology of the OMU for metagenomes and the OTU for microbes
Source: BMC Bioinformatics. 2024 Mar 29;25:137. doi: 10.1186/s12859-023-05591-8 (PMC10979563; doi:10.1186/s12859-023-05591-8)
Supplement: Supplementary file 1 — Additional file 1. Table S1–S22. [file 12859_2023_5591_MOESM1_ESM.pdf]

Online Supplementary Information:

Ma ZS (2023) Towards a unified medical microbiome ecology of the OMU for metagenomes and OTU for microbes. *BMC Bioinformatics.*

Table S1. The parameters of Sloan (2006, 2007) neutral model fitted to metagenomic gene (MG) abundance data of the four datasets (each cohort is treated as both the source & destination communities in Sloan model)

Disease	Treatments	*N***	*m*	R^2	Total***	Below Neutral*	Neutral*	Above Neutral*
Gut	Stool	33943867.4	0.004	0.145	5838354	723947 (12.4%)	5198 (0.09%)	5109209 (87.5%)
Obesity	Lean	24809143.8	0.012	0.218	7119509	1336778 (18.8%)	12947 (0.18%)	5769784 (81.0%)
	Overweight	25790382.0	0.012	0.223	6883584	1321298 (19.2%)	2767 (0.04%)	5559519 (80.8%)
Type-II Diabetes	Healthy	12156497.9	0.015	0.306	4420933	617898 (14.0%)	1520 (0.03%)	3801515 (86.0%)
	Disease	11678477.1	0.012	0.322	4664260	621812 (13.3%)	4146 (0.09%)	4038302 (86.6%)
IBD	Healthy	24555484.4	0.017	0.309	6161952	1177039 (19.1%)	5601 (0.09%)	4979312 (80.8%)
	Disease	22377602.4	0.012	0.281	5966172	1047155 (17.6%)	2985 (0.05%)	4916032 (82.4%)
Mean		22187350.7	0.012	0.257	5864966.3	977989.6 (16.3%)	5023.4(0.1%)	4881953.3 (83.6%)
Std. Err.		2987155.1	0.002	0.024	385373.6	120718.1	1426.3	275539.2

*The numbers enclosed in parentheses are the percentage of each category of MG out of total number of MGs.
***N* is the average total gene abundance (total *reads*) per metagenome sample; *m* is the migration probability.
***Total is the total number of genes in the metacommunity of metagenomes, or in the assembly of metagenomes.

Table S2. The parameters of Sloan (2006, 2007) neutral model for Type-II MFGC (metagenome functional gene cluster) with each cohort being treated as both source and destination communities of Sloan model

Database	Disease	Treatments	*N***	*m*	R^2	Total	*Below Neutral	*Neutral	*Above Neutral
EggNOG	Gut	Stool	28211687.3	0.005	0.812	337	47(13.9)	192(57)	98(29.1)
	Obesity	Lean	21339923.5	0.008	0.824	354	49(13.8)	198(55.9)	107(30.2)
		Overweight	22096627.5	0.008	0.836	354	47(13.3)	208(58.8)	99(28)
	Type II Diabetes	Healthy	10254615.5	0.018	0.867	327	31(9.5)	230(70.3)	66(20.2)
		Disease	9854123.6	0.012	0.881	320	36(11.3)	220(68.8)	64(20)
	IBD	Healthy	21068367.4	0.014	0.818	348	47(13.5)	212(60.9)	89(25.6)
		Disease	19269709.8	0.010	0.886	350	36(10.3)	227(64.9)	87(24.9)
Mean			18870722.1	0.011	0.846	341.4	41.9(12.2)	212.4(62.4)	87.1(25.4)
Standard Error			2508472.5	0.002	0.012	5.2	2.7	5.4	6.2
KEGG	Gut	Stool	33943867.4	0.013	0.787	273	76(27.8)	107(39.2)	90(33)
	Obesity	Lean	24809143.8	0.018	0.794	287	56(19.5)	146(50.9)	85(29.6)
		Overweight	25790382.0	0.018	0.783	283	60(21.2)	129(45.6)	94(33.2)
	Type II Diabetes	Healthy	12156497.9	0.033	0.774	255	55(21.6)	127(49.8)	73(28.6)
		Disease	11678477.1	0.023	0.805	262	52(19.8)	139(53.1)	71(27.1)
	IBD	Healthy	24555484.4	0.026	0.815	280	47(16.8)	148(52.9)	85(30.4)
		Disease	22377602.4	0.017	0.826	276	48(17.4)	149(54)	79(28.6)
Mean			22187350.7	0.021	0.798	273.7	56.3(20.6)	135(49.4)	82.4(30.1)
Standard Error			2987155.1	0.003	0.007	4.4	3.7	5.7	3.2

* Numbers inside parentheses are the percentages of MFGC in each category.

** ***N*** is the average total gene abundance (total reads) per metagenome sample.

*** Total is the total number of MFGCs

Since the definition of Type-I MFGC ignored the metagenomic gene abundance within MFGC cluster, it cannot fit the neutral model. Therefore, only the results for Type-II MFGC are computed in this study.

Table S3. The parameters of Sloan (2006, 2007) neutral model for Type-II MFGC with the healthy as source and diseased as destination community in Sloan model

Database	Treatments	Database	*N*	*m*	R^2	Total	Below Neutral	Neutral	Above Neutral
Obesity	Lean vs. Overweight	EggNOG	21068367.4	0.019	0.843	341	47(13.8)	210(61.6)	84(24.6)
		KEGG	24555484.4	0.025	0.766	268	51(19)	129(48.1)	88(32.8)
Type-II Diabetes	Healthy vs. Disease	EggNOG	10254615.4	0.012	0.847	310	35(11.3)	196(63.2)	79(25.5)
		KEGG	12156497.9	0.028	0.770	246	49(19.9)	121(49.2)	76(30.9)
IBD	Healthy vs. UC	EggNOG	21339923.5	0.011	0.811	351	53(15.1)	199(56.7)	99(28.2)
		KEGG	24809143.8	0.022	0.758	279	61(21.9)	132(47.3)	86(30.8)
Mean across treatments		EggNOG	17554302.1	0.014	0.834	334	45(13.4)	201.7(60.5)	87.3(26.1)
Standard Error			3650685.101	0.003	0.011	12.342	5.3	4.3	6
Mean across treatments		KEGG	20507042.03	0.025	0.765	264.333	53.7(20.3)	127.3(48.2)	83.3(31.5)
Standard Error			4175914.122	0.002	0.004	9.701	3.7	3.3	3.7

* Numbers inside parentheses are the percentages of MFGC in each category.
** ***N*** is the average total gene abundance (total reads) per metagenome sample.
*** Total is the total number of MFGCs

Table S4 (MS-Excel Table). The lists of Type-II MFGCs in each of the three categories: below neutral, neutral and above neutral (Excel Table) for each metagenome treatment.

Table S5. The *P*-value of the randomization test for the differences (in Sloan neutral model parameters for the MFGCs, listed in Table S2) between the healthy control and diseased treatments

Database	Treatments	Database	*N*	*m*	R^2	Total	*Below Neutral	*Neutral	*Above Neutral
Obesity	Lean vs. Overweight	EggNOG	0.594	0.954	0.75	0.822	0.577	0.543	0.633
		KEGG	0.55	0.945	0.723	0.25	0.563	0.092	0.51
Type II Diabetes	Healthy vs. Disease	EggNOG	0.312	0.079	0.476	0.263	0.303	0.366	0.828
		KEGG	0.31	0.079	0.327	0.237	0.61	0.282	0.777
IBD	Healthy vs. UC	EggNOG	0.219	0.119	0.000	0.516	0.034	0.216	0.842
		KEGG	0.215	0.011	0.634	0.381	0.813	0.908	0.461

*The test was done with the number of MFGCs, rather that with the percentage.

Table S6. The proportions of neutral and non-neutral MFGCs in the core and periphery, respectively, of the CPNs (core/periphery networks) of human gut metagenomes

Study	Treatments		Below Neutral	Neutral	Above Neutral
**Type-II MFGC (eggNOG)**					
Gut	Stool	Core	11.5% (21/183)	55.7% (102/183)	24.6% (45/183)
		Periphery	13.8% (21/152)	51.3% (78/152)	29.6% (45/152)
Obesity	Lean	Core	14% (23/164)	52.4% (86/164)	31.1% (51/164)
		Periphery	11.4% (20/176)	58.5% (103/176)	27.3% (48/176)
	Overweight	Core	13.8% (25/181)	60.2% (109/181)	23.2% (42/181)
		Periphery	11.4% (19/167)	55.1% (92/167)	31.1% (52/167)
Type-II Diabetes	Healthy	Core	9.3% (15/161)	66.5% (107/161)	14.9% (24/161)
		Periphery	6.3% (10/159)	61% (97/159)	23.3% (37/159)
	Disease	Core	8.3% (14/169)	63.3% (107/169)	13.6% (23/169)
		Periphery	10.3% (15/146)	60.3% (88/146)	21.9% (32/146)
IBD	Healthy	Core	10.4% (17/163)	62% (101/163)	23.9% (39/163)
		Periphery	14.4% (26/180)	55.6% (100/180)	25.6% (46/180)
	Disease	Core	12.6% (21/167)	65.3% (109/167)	18.6% (31/167)
		Periphery	7.2% (13/180)	58.9% (106/180)	30% (54/180)
**Average**		Core	11.4%	60.8%	21.4%
		Periphery	10.7%	57.2%	27.0%
**Type-II MFGC (KEGG)**					
Gut	Stool	Core	24.1% (34/141)	34.8% (49/141)	27% (38/141)
		Periphery	22.5% (29/129)	35.7% (46/129)	30.2% (39/129)
Obesity	Lean	Core	13.5% (13/96)	52.1% (50/96)	25% (24/96)
		Periphery	19.1% (27/141)	45.4% (64/141)	30.5% (43/141)
	Overweight	Core	17.5% (21/120)	48.3% (58/120)	21.7% (26/120)
		Periphery	20.6% (29/141)	35.5% (50/141)	37.6% (53/141)
Type-II Diabetes	Healthy	Core	16.1% (15/93)	38.7% (36/93)	23.7% (22/93)
		Periphery	20.3% (28/138)	38.4% (53/138)	25.4% (35/138)
	Disease	Core	16.5% (19/115)	33% (38/115)	33% (38/115)
		Periphery	16.5% (22/133)	50.4% (67/133)	16.5% (22/133)
IBD	Healthy	Core	12% (11/92)	43.5% (40/92)	31.5% (29/92)
		Periphery	14.6% (21/144)	47.2% (68/144)	29.2% (42/144)
	Disease	Core	12.3% (13/106)	47.2% (50/106)	26.4% (28/106)
		Periphery	15.8% (21/133)	48.1% (64/133)	24.8% (33/133)
**Average**		Core	16.0%	42.5%	26.9%
		Periphery	18.5%	42.9%	27.7%

Table S7. Randomization test for the proportions (see Table S6) of neutral and non-neutral MFGCs in the core *vs*. periphery between the healthy and diseased samples

Study	Treatments	Core			Periphery		
		**Below**	**Neutral**	**Above**	**Below**	**Neutral**	**Above**
			**Type-II MFGC (KEGG)**				
Obesity	Lean vs. Overweight	0.30	0.56	0.34	0.73	0.17	0.12
Type-2 Diabetes	Healthy vs. Disease	0.91	0.15	0.01	0.29	0.07	0.03
IBD	Healthy vs. Disease	0.91	0.53	0.26	0.73	0.83	0.25
			**Type-II MFGC (eggNOG)**				
Obesity	Lean vs. Overweight	0.90	0.24	0.15	0.98	0.36	0.27
Type 2 diabetes	Healthy vs. Disease	0.72	0.68	0.61	0.11	0.80	0.77
IBD	Healthy vs. Disease	0.63	0.62	0.10	0.05	0.55	0.15

Table S8A. The number of various *trios* in the class "*Trios without MAO handle*" in the MFGC networks (with FDR adjustment)

Microbiomes & Associated Disease Treatments		Trios with MAO: Type-1 (+ − −) Type-1A	Type-1B	Σ	Type-2 (+ + −) Type-2A	Type-2B	Σ	Type-3 (− − −)	Type-4 (+ + +)	Σ	Trios without MAO: − − −	+ − −	+ + −	+ + +	Σ
**MFGC Type-I (KEGG)**															
Stool	Stool	246	210	456	1	0	1	0	105	562	5	2515	23	1865	4408
Obesity	Lean	133	93	226	0	0	0	0	47	273	0	1255	0	639	1894
	Overweight	114	42	156	0	0	0	0	54	210	0	948	1	441	1390
Type 2 diabetes	Healthy	222	104	326	0	0	0	0	132	458	0	2548	2	975	3525
	Disease	77	88	165	0	0	0	0	24	189	0	757	0	332	1089
IBD	Healthy	84	52	136	0	0	0	0	33	169	0	592	0	299	891
	Disease	35	29	64	0	0	0	0	13	77	0	387	0	298	685
**MFGC Type-II (KEGG)**															
Stool	Stool	758	3055	3813	0	0	0	0	75	3888	7	21928	52	55059	77046
Obesity	Lean	102	113	215	0	0	0	1	20	236	1	1138	0	814	1953
	Overweight	151	339	490	0	0	0	0	12	502	1	2270	8	1987	4266
Type 2 diabetes	Healthy	67	126	193	0	0	0	0	16	209	0	1087	0	1210	2297
	Disease	87	78	165	0	0	0	0	36	201	0	944	0	2475	3419
IBD	Healthy	83	107	190	0	0	0	0	10	200	0	694	0	428	1122
	Disease	27	21	48	0	0	0	0	9	57	0	352	1	389	742
**MFGC Type-I (eggNOG)**															
Stool	Stool	1230	635	1865	2	40	42	13	1302	3222	630	95420	1494	52810	150354
Obesity	Lean	835	375	1210	3	4	7	3	705	1925	44	57956	265	30615	88880
	Overweight	631	216	847	1	4	5	2	638	1492	86	59565	283	34261	94195
Type 2 diabetes	Healthy	468	231	699	0	5	5	2	491	1197	44	106124	113	56048	162329
	Disease	249	76	325	0	3	3	0	387	715	4	57571	30	29912	87517
IBD	Healthy	627	226	853	0	0	0	0	406	1259	1	59124	8	28810	87943
	Disease	797	237	1034	0	0	0	0	834	1868	9	35665	41	21857	57572
**MFGC Type-II (eggNOG)**															
Stool	Stool	2722	4413	7135	2	18	20	8	465	7628	261	174635	1418	138256	314570
Obesity	Lean	196	225	421	0	2	2	1	61	485	42	47892	182	26874	74990
	Overweight	675	623	1298	1	8	9	12	204	1523	115	91182	534	47157	138988
Type 2 diabetes	Healthy	609	557	1166	0	0	0	0	170	1336	13	51901	89	33122	85125
	Disease	772	410	1182	0	0	0	0	466	1648	7	41795	21	25128	66951
IBD	Healthy	468	208	676	0	0	0	0	324	1000	2	35395	22	21748	57167
	Disease	364	225	589	0	0	0	0	165	754	9	19957	25	20112	40103

Table S8B. Randomization test for the number of various *trios* in the class "*Trios without MAO handle*" in the MFGC networks

Microbiomes & Associated Disease Treatments		Trios with MAO 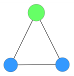									Trios without MAO 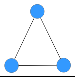				
		+ – – Type-1			+ + – Type-2			Type-3 –	Type-4 +		–	+	+	+	
		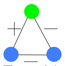 Type-1A	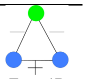 Type-1B	Σ	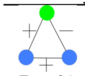 Type-2A	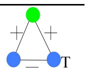 Type-2B	Σ	–	+	Σ	–	–	+	+	Σ
								–	+		–	–	–	+	
**MFGC Type-I (KEGG)**															
Obesity	Lean vs. Overweight	0.86	0.47	0.63	1.00	1.00	1.00	1.00	0.84	0.73	1.00	0.66	0.80	0.59	0.63
Type 2 diabetes	Healthy vs. Disease	0.16	0.83	0.32	1.00	1.00	1.00	1.00	0.05	0.18	1.00	0.06	0.10	0.08	0.06
IBD	Healthy vs. Disease	0.28	0.63	0.41	1.00	1.00	1.00	1.00	0.21	0.34	1.00	0.36	1.00	1.00	0.63
**MFGC Type-II (KEGG)**															
Obesity	Lean vs. Overweight	0.50	0.14	0.23	1.00	1.00	1.00	0.36	0.45	0.27	1.00	0.28	0.03	0.29	0.27
Type 2 diabetes	Healthy vs. Disease	0.70	0.59	0.83	1.00	1.00	1.00	1.00	0.26	0.98	1.00	0.75	1.00	0.45	0.58
IBD	Healthy vs. Disease	0.04	0.07	0.06	1.00	1.00	1.00	1.00	0.81	0.07	1.00	0.11	0.15	0.80	0.32
**MFGC Type-I (eggNOG)**															
Obesity	Lean vs. Overweight	0.76	0.44	0.68	0.59	1.00	0.85	0.86	0.85	0.70	0.34	0.94	0.90	0.68	0.89
Type 2 diabetes	Healthy vs. Disease	0.35	0.17	0.22	1.00	0.66	0.67	0.47	0.75	0.46	0.00	0.12	0.03	0.07	0.11
IBD	Healthy vs. Disease	0.70	0.98	0.72	1.00	1.00	1.00	1.00	0.50	0.59	0.23	0.21	0.12	0.39	0.24
**MFGC Type-II (eggNOG)**															
Obesity	Lean vs. Overweight	0.11	0.11	0.10	0.75	0.24	0.29	0.09	0.10	0.09	0.20	0.22	0.05	0.27	0.23
Type 2 diabetes	Healthy vs. Disease	0.70	0.56	0.98	1.00	1.00	1.00	1.00	0.26	0.72	0.32	0.49	0.03	0.24	0.31
IBD	Healthy vs. Disease	0.68	0.93	0.81	1.00	1.00	1.00	1.00	0.28	0.62	0.27	0.08	0.81	0.82	0.25

Table S8C. The number of “Trios with MAO handle” in MFGC networks with FDR control

Microbiomes & Associated Disease Treatments		Single-Link MAO 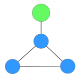			Double-Link MAO 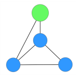				Triple-Link MAO 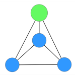				
		–	+	Σ	– –	+ –	+ +	Σ	– – –	+ – –	+ + –	+ + +	Σ
**MFGC Type-I (KEGG)**													
Stool	Stool	231	553	784	232	387	377	996	501	829	563	191	2084
Obesity	Lean	141	92	233	190	306	135	631	185	422	299	62	968
	Overweight	80	94	174	54	127	73	254	49	242	282	88	661
Type 2 diabetes	Healthy	44	73	117	121	307	162	590	324	948	1059	385	2716
	Disease	100	80	180	111	157	49	317	137	209	133	21	500
IBD	Healthy	89	49	138	71	121	43	235	83	189	138	25	435
	Disease	102	60	162	33	32	11	76	20	33	20	2	75
**MFGC Type-II (KEGG)**													
Stool	Stool	903	94	997	6489	1441	115	8045	47206	17784	2424	195	67609
Obesity	Lean	261	65	326	382	301	54	737	231	390	152	18	791
	Overweight	357	135	492	632	419	48	1099	1142	1004	208	10	2364
Type 2 diabetes	Healthy	458	145	603	505	304	98	907	232	181	102	16	531
	Disease	888	67	955	400	237	146	783	125	256	264	64	709
IBD	Healthy	91	18	109	125	99	19	243	189	335	127	10	661
	Disease	139	42	181	64	78	12	154	8	34	33	7	82
**MFGC Type-I (eggNOG)**													
Stool	Stool	17985	18666	36651	10707	14279	18711	43697	4322	9815	18071	17010	49218
Obesity	Lean	12339	14365	26704	7995	14815	9989	32799	2088	6345	6889	3883	19205
	Overweight	12849	22015	34864	4652	12403	11575	28630	866	3563	4786	3344	12559
Type 2 diabetes	Healthy	22947	28546	51493	3152	8804	10167	22123	678	2026	4666	3736	11106
	Disease	11564	10550	22114	1286	2879	3467	7632	101	542	1606	2200	4449
IBD	Healthy	13307	15747	29054	5241	13429	7731	26401	912	3935	5000	1950	11797
	Disease	6423	7928	14351	2987	7741	9419	20147	757	3479	6420	5159	15815
**MFGC Type-II (eggNOG)**													
Stool	Stool	9015	4214	13229	35418	25064	5033	65515	102042	93945	29888	3339	229214
Obesity	Lean	8558	10960	19518	2447	4043	1925	8415	759	1017	741	160	2677
	Overweight	27996	17063	45059	11605	15045	4756	31406	3773	6819	4267	877	15736
Type 2 diabetes	Healthy	11598	10845	22443	7472	7108	1965	16545	4651	7475	3528	554	16208
	Disease	8164	6880	15044	6185	11777	7204	25166	2129	6436	7353	3298	19216
IBD	Healthy	6247	9398	15645	2979	8649	6773	18401	870	3193	4287	2141	10491
	Disease	3291	3478	6769	1944	3461	1657	7062	866	2184	2005	619	5674

Table S8D. Randomization test for the number of “Trios with MAO handle” in the MFGC networks

Microbiomes & Associated Disease Treatments		Single-Link MAO 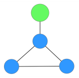			Double-Link MAO 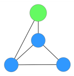				Triple-Link MAO 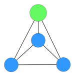				
		−	+	Σ	−<br>−	+<br>−	+<br>+	Σ	−<br>−<br>−	+<br>−<br>−	+<br>+<br>−	+<br>+<br>+	Σ
**MFGC Type-I (KEGG)**													
Obesity	Lean vs. Overweight	0.65	0.95	0.72	0.37	0.20	0.29	0.24	0.51	0.61	0.97	0.69	0.71
Type 2 diabetes	Healthy vs. Disease	0.41	0.92	0.58	0.93	0.32	0.15	0.33	0.38	0.08	0.04	0.01	0.06
IBD	Healthy vs. Disease	0.85	0.76	0.81	0.68	0.10	0.00	0.17	0.53	0.21	0.08	0.05	0.23
**MFGC Type-II (KEGG)**													
Obesity	Lean vs. Overweight	0.57	0.23	0.47	0.49	0.58	0.88	0.51	0.20	0.32	0.67	0.52	0.31
Type 2 diabetes	Healthy vs. Disease	0.51	0.33	0.67	0.92	0.64	0.53	0.91	0.65	0.80	0.30	0.30	0.79
IBD	Healthy vs. Disease	0.51	0.51	0.48	0.37	0.66	0.75	0.44	0.04	0.00	0.08	0.74	0.01
**MFGC Type-I (eggNOG)**													
Obesity	Lean vs. Overweight	0.94	0.35	0.60	0.25	0.72	0.77	0.75	0.41	0.70	0.80	0.86	0.74
Type 2 diabetes	Healthy vs. Disease	0.10	0.14	0.07	0.33	0.12	0.18	0.12	0.15	0.22	0.36	0.67	0.37
IBD	Healthy vs. Disease	0.11	0.11	0.11	0.32	0.34	0.83	0.68	0.93	0.92	0.83	0.60	0.78
**MFGC Type-II (eggNOG)**													
Obesity	Lean vs. Overweight	0.07	0.51	0.19	0.02	0.06	0.21	0.05	0.12	0.11	0.13	0.14	0.10
Type 2 diabetes	Healthy vs. Disease	0.55	0.30	0.36	0.72	0.40	0.32	0.45	0.23	0.80	0.48	0.20	0.81
IBD	Healthy vs. Disease	0.08	0.07	0.05	0.50	0.03	0.00	0.01	1.00	0.67	0.41	0.08	0.51

Table S9A. The P/N (positive to negative links) ratios in the MFGC (Metagenome Functional Gene Cluster) networks with FDR adjustment

Microbiome	Treatments	Positive Links (+)	Negative Links (–)	P/N (+/–) Ratio
		**MFGC Type-I (KEGG)**		
Stool	Stool	1019	561	1.816
Obesity	Lean	532	340	1.565
	Overweight	429	307	1.397
Type 2 diabetes	Healthy	588	415	1.417
	Disease	422	254	1.661
IBD	Healthy	378	234	1.615
	Disease	438	242	1.810
		**MFGC Type-II (KEGG)**		
Stool	Stool	4625	1039	4.451
Obesity	Lean	623	265	2.351
	Overweight	1020	419	2.434
Type 2 diabetes	Healthy	838	283	2.961
	Disease	1070	214	5.000
IBD	Healthy	503	208	2.418
	Disease	568	163	3.485
		**MFGC Type-I (eggNOG)**		
Stool	Stool	5818	4357	1.335
Obesity	Lean	4471	3202	1.396
	Overweight	4777	3287	1.453
Type 2 diabetes	Healthy	5218	3733	1.398
	Disease	3964	2786	1.423
IBD	Healthy	4049	3069	1.319
	Disease	4046	2707	1.495
		**MFGC Type-II (eggNOG)**		
Stool	Stool	8396	4454	1.885
Obesity	Lean	4171	2631	1.585
	Overweight	5638	3844	1.467
Type 2 diabetes	Healthy	4284	2818	1.520
	Disease	3802	2271	1.674
IBD	Healthy	3665	1979	1.852
	Disease	3831	1829	2.095

Table S9B. Randomization test for the P/N (positive to negative links) ratios in the MFGC (Metagenome Functional Gene Cluster) networks with FDR adjustment

Microbiome	Treatments	Positive Links (+)	Negative Links (–)	P/N (+/–) Ratio
		**MFGC Type-I (KEGG)**		
Obesity	Lean vs. Overweight	0.40	0.84	0.30
Type 2 diabetes	Healthy vs. Disease	0.26	0.12	0.20
IBD	Healthy vs. Disease	0.63	0.92	0.45
		**MFGC Type-II (KEGG)**		
Obesity	Lean vs. Overweight	0.14	0.15	0.89
Type 2 diabetes	Healthy vs. Disease	0.59	0.32	0.02
IBD	Healthy vs. Disease	0.63	0.36	0.09
		**MFGC Type-I (eggNOG)**		
Obesity	Lean vs. Overweight	0.62	0.91	0.61
Type 2 diabetes	Healthy vs. Disease	0.10	0.19	0.78
IBD	Healthy vs. Disease	1.00	0.56	0.18
		**MFGC Type-II (eggNOG)**		
Obesity	Lean vs. Overweight	0.13	0.10	0.43
Type 2 diabetes	Healthy vs. Disease	0.31	0.20	0.42
IBD	Healthy vs. Disease	0.78	0.65	0.43

Table S10 (MS-Excel Table). The list of Core/Periphery nodes from the MFGC (metagenome functional gene cluster) networks

Table S11A. The core/periphery and nested structures in the MFGC (Metagenome Functional Gene Cluster) networks with FDR adjustment

Microbiome	Treatments	ρ	Ratio of C/(C+P)	Density Matrix			P/N Ratio				Nestedness (S)
				B11	B12(21)	B22	Whole	Core	Periph-ery	C-P	
**MFGC Type-I (KEGG)**											
Stool	Stool	0.215	0.463	0.138	0.028	0.018	1.816	1.499	5.036	2.020	0.115
Obesity	Lean	0.246	0.407	0.135	0.017	0.011	1.565	1.287	4.316	1.784	0.092
	Overweight	0.234	0.433	0.122	0.016	0.009	1.397	1.253	4.077	1.356	0.100
Type 2 diabetes	Healthy	0.304	0.380	0.197	0.023	0.014	1.417	1.035	5.316	1.976	0.126
	Disease	0.238	0.378	0.133	0.015	0.013	1.661	1.087	8.818	2.383	0.078
IBD	Healthy	0.212	0.375	0.111	0.016	0.008	1.615	1.377	3.294	1.727	0.069
	Disease	0.187	0.407	0.092	0.015	0.009	1.810	1.634	4.000	1.667	0.058
**MFGC Type-II (KEGG)**											
Stool	Stool	0.379	0.522	0.381	0.085	0.043	4.451	4.081	10.484	4.776	0.305
Obesity	Lean	0.220	0.405	0.119	0.018	0.011	2.351	1.729	6.133	3.667	0.080
	Overweight	0.216	0.460	0.127	0.023	0.014	2.434	1.867	14.222	3.213	0.108
Type 2 diabetes	Healthy	0.221	0.403	0.144	0.027	0.017	2.961	2.015	25.333	3.767	0.090
	Disease	0.214	0.464	0.124	0.022	0.016	5.000	4.056	14.556	6.500	0.091
IBD	Healthy	0.181	0.390	0.093	0.015	0.012	2.418	1.506	7.200	4.324	0.055
	Disease	0.162	0.444	0.078	0.014	0.012	3.485	2.273	9.500	8.238	0.051
**MFGC Type-I (eggNOG)**											
Stool	Stool	0.289	0.545	0.355	0.123	0.080	1.335	1.232	2.898	1.270	0.325
Obesity	Lean	0.271	0.480	0.300	0.099	0.049	1.396	1.164	2.534	1.557	0.249
	Overweight	0.174	0.624	0.059	0.136	0.329	1.453	2.009	1.277	1.428	0.251
Type 2 diabetes	Healthy	0.348	0.512	0.394	0.113	0.057	1.398	1.277	2.356	1.470	0.328
	Disease	0.302	0.502	0.318	0.091	0.052	1.423	1.225	2.099	1.675	0.253
IBD	Healthy	0.268	0.488	0.277	0.087	0.043	1.319	1.163	2.051	1.434	0.236
	Disease	0.201	0.510	0.221	0.088	0.051	1.495	1.469	1.594	1.503	0.203
**MFGC Type-II (eggNOG)**											
Stool	Stool	0.363	0.546	0.464	0.145	0.095	1.885	1.513	5.594	2.322	0.391
Obesity	Lean	0.263	0.482	0.273	0.086	0.044	1.585	1.278	3.383	1.830	0.226
	Overweight	0.269	0.520	0.318	0.118	0.053	1.467	1.172	4.000	1.719	0.290
Type 2 diabetes	Healthy	0.297	0.503	0.316	0.091	0.055	1.520	1.274	3.389	1.689	0.234
	Disease	0.277	0.537	0.266	0.074	0.044	1.674	1.295	8.592	2.168	0.213
IBD	Healthy	0.277	0.475	0.248	0.058	0.041	1.852	1.325	6.151	2.567	0.182
	Disease	0.193	0.481	0.197	0.077	0.039	2.095	1.883	3.253	2.138	0.162

Table S11B. Randomization test for the core/periphery and nested structures in the MFGC (Metagenome Functional Gene Cluster) with FDR adjustment

Microbiome	Treatments	ρ	Ratio of C/(C+P)	Density Matrix			P/N Ratio				Nestedness (S)
				B11	B12(21)	B22	Whole	Core	Periph-ery	C-P	
				**MFGC Type-I (KEGG)**							
Obesity	Lean vs. Overweight	0.78	0.65	0.70	0.86	0.32	0.30	0.82	0.91	0.25	0.74
Type 2 diabetes	Healthy vs. Disease	0.24	0.96	0.17	0.11	0.58	0.20	0.66	0.38	0.49	0.02
IBD	Healthy vs. Disease	0.53	0.57	0.60	0.80	0.60	0.45	0.33	0.72	0.88	0.36
				**MFGC Type-II (KEGG)**							
Obesity	Lean vs. Overweight	0.95	0.26	0.80	0.27	0.27	0.89	0.73	0.33	0.66	0.17
Type 2 diabetes	Healthy vs. Disease	0.83	0.23	0.61	0.58	0.73	0.02	0.21	0.33	0.12	0.98
IBD	Healthy vs. Disease	0.45	0.30	0.45	0.81	1.00	0.09	0.14	0.87	0.08	0.68
				**MFGC Type-I (eggNOG)**							
Obesity	Lean vs. Overweight	0.06	0.00	0.04	0.08	0.03	0.61	0.09	0.17	0.49	0.95
Type 2 diabetes	Healthy vs. Disease	0.47	0.84	0.40	0.43	0.73	0.78	0.64	0.71	0.21	0.12
IBD	Healthy vs. Disease	0.17	0.61	0.32	0.94	0.55	0.18	0.19	0.46	0.65	0.37
				**MFGC Type-II (eggNOG)**							
Obesity	Lean vs. Overweight	0.86	0.47	0.51	0.29	0.56	0.43	0.40	0.72	0.75	0.28
Type 2 diabetes	Healthy vs. Disease	0.63	0.38	0.37	0.27	0.36	0.42	0.90	0.00	0.14	0.45
IBD	Healthy vs. Disease	0.06	0.88	0.29	0.25	0.87	0.43	0.24	0.12	0.39	0.44

Table S12. The shared MFGC analysis between the healthy and diseased treatments

Microbiome	Treatments	Reads randomization (A1)*			Samples randomization (A2)*		
		Core	Periphery	Total	Core	Periphery	Total
**MFGC Type-I (KEGG)**							
Obesity	Lean vs. Overweight	0.821	0.842	0.738	0.800	0.828	0.713
Type 2 diabetes	Healthy vs. Disease	0.387	0.716	0.459	0.361	0.735	0.458
IBD	Healthy vs. Disease	0.933	0.653	0.588	0.907	0.684	0.579
**MFGC Type-II (KEGG)**							
Obesity	Lean vs. Overweight	0.289	0.000	0.000	1.000	0.707	0.741
Type 2 diabetes	Healthy vs. Disease	0.286	0.000	0.000	1.000	0.547	0.444
IBD	Healthy vs. Disease	0.000	0.000	0.000	0.607	0.791	0.563
**MFGC Type-I (eggNOG)**							
Obesity	Lean vs. Overweight	0.928	0.903	0.762	0.939	0.920	0.791
Type 2 diabetes	Healthy vs. Disease	0.185	0.049	0.003	0.281	0.155	0.065
IBD	Healthy vs. Disease	0.281	0.394	0.121	0.313	0.445	0.119
**MFGC Type-II (eggNOG)**							
Obesity	Lean vs. Overweight	0.000	0.069	0.000	0.447	0.982	0.757
Type 2 diabetes	Healthy vs. Disease	0.000	0.000	0.000	0.141	0.200	0.060
IBD	Healthy vs. Disease	0.000	0.000	0.000	0.106	0.871	0.134

Algorithm **A1** is randomization reassignments (remix) of all reads across samples and MFGCs.
Algorithm **A2** is randomization reassignments (remix) of all samples only.
See Ma *et al*. (2019) ISME Journal or Ma (2020) for detailed introduction of **A1** & **A2**.

Table S13A. Statistical properties of the high-salience skeletons in the MFGC (Metagenome Functional Gene Cluster) networks with FDR adjustment

**Microbiome**	**Treatments**	**Statistics of HSS**						Assortativity
		Links (%)	Max	Mean	Std. Err	Skewness	Kurtosis	r_{HSS}
**MFGC Type-I (KEGG)**								
Stool	Stool	4.81	1.000	0.008	0.058	11.646	160.244	-0.004
Obesity	Lean	12.91	0.951	0.009	0.067	11.320	140.646	-0.004
	Overweight	12.65	0.952	0.010	0.071	10.856	127.743	-0.005
Type 2 diabetes	Healthy	14.44	0.948	0.009	0.067	11.132	136.021	-0.005
	Disease	10.62	0.962	0.010	0.073	10.521	118.659	-0.005
IBD	Healthy	42.67	0.773	0.009	0.050	12.083	162.225	-0.005
	Disease	23.77	0.887	0.009	0.058	11.490	149.853	-0.004
**MFGC Type-II (KEGG)**								
Stool	Stool	14.88	1.000	0.007	0.039	13.183	237.117	-0.004
Obesity	Lean	8.16	0.975	0.008	0.069	11.363	140.230	-0.004
	Overweight	5.75	0.992	0.008	0.061	11.996	164.040	-0.004
Type 2 diabetes	Healthy	8.50	0.978	0.009	0.060	10.933	140.458	-0.004
	Disease	7.39	0.984	0.008	0.060	11.422	150.657	-0.004
IBD	Healthy	13.26	0.945	0.008	0.067	11.139	134.491	-0.004
	Disease	7.53	0.975	0.008	0.065	10.790	132.653	-0.004
**MFGC Type-I (eggNOG)**								
Stool	Stool	16.28	0.961	0.006	0.027	11.855	225.866	-0.003
Obesity	Lean	12.62	1.000	0.006	0.034	16.258	375.832	-0.003
	Overweight	12.94	1.000	0.006	0.032	16.007	380.409	-0.003
Type 2 diabetes	Healthy	16.95	1.000	0.006	0.033	14.725	306.032	-0.003
	Disease	13.74	1.000	0.006	0.037	15.369	317.375	-0.003
IBD	Healthy	12.04	1.000	0.006	0.036	16.179	350.718	-0.003
	Disease	11.36	1.000	0.006	0.031	15.516	372.027	-0.003
**MFGC Type-II (eggNOG)**								
Stool	Stool	19.61	0.970	0.006	0.026	12.263	223.489	-0.003
Obesity	Lean	11.39	1.000	0.006	0.036	15.767	340.604	-0.003
	Overweight	15.02	1.000	0.006	0.031	16.768	405.758	-0.003
Type 2 diabetes	Healthy	13.73	1.000	0.006	0.032	13.557	272.117	-0.003
	Disease	13.44	0.994	0.006	0.035	15.088	326.274	-0.003
IBD	Healthy	10.75	0.994	0.006	0.036	14.806	298.504	-0.003
	Disease	10.56	0.994	0.006	0.031	13.479	283.784	-0.003

Table S13B. Randomization test for the statistical properties of the high-salience skeletons in the MFGC (Metagenome Functional Gene Cluster) networks with FDR adjustment

**Microbiome**	**Treatments**	**Statistics of HSS**						**Assortativity**
		Links (%)	Max	Mean	Std. Err	Skewness	Kurtosis	r_{HSS}
		**MFGC Type-I (KEGG)**						
Obesity	Lean vs. Overweight	0.99	0.99	0.27	0.60	0.42	0.44	0.02
Type 2 diabetes	Healthy vs. Disease	0.60	0.77	0.22	0.29	0.26	0.23	1.00
IBD	Healthy vs. Disease	0.09	0.07	1.00	0.22	0.30	0.43	0.07
		**MFGC Type-II (KEGG)**						
Obesity	Lean vs. Overweight	0.76	0.71	1.00	0.07	0.20	0.12	1.00
Type 2 diabetes	Healthy vs. Disease	0.75	0.78	0.06	1.00	0.43	0.60	1.00
IBD	Healthy vs. Disease	0.62	0.66	1.00	0.75	0.50	0.92	1.00
		**MFGC Type-I (eggNOG)**						
Obesity	Lean vs. Overweight	0.84	1.00	1.00	0.33	0.82	0.97	1.00
Type 2 diabetes	Healthy vs. Disease	0.22	1.00	1.00	0.14	0.53	0.78	1.00
IBD	Healthy vs. Disease	0.73	1.00	1.00	0.04	0.40	0.55	1.00
		**MFGC Type-II (eggNOG)**						
Obesity	Lean vs. Overweight	0.18	1.00	1.00	0.06	0.25	0.05	1.00
Type 2 diabetes	Healthy vs. Disease	0.89	0.28	1.00	0.27	0.12	0.08	1.00
IBD	Healthy vs. Disease	0.94	1.00	1.00	0.03	0.12	0.65	1.00

Table S14. The index for MF (Metagenome Function, based on eggNOG database) and MP (Metagenome Pathway, based on KEGG database) for building MF/MP networks, each node in the network represents one function (eggNOG) or one pathway (KEGG)

**eggNOG**	Nucleotide transport and metabolism;<br>Replication, recombination and repair;<br>Intracellular trafficking, secretion, and vesicular transport;<br>Translation, ribosomal structure and biogenesis;<br>Transcription;<br>Chromatin structure and dynamics;<br>Cell cycle control, cell division, chromosome partitioning;<br>Defense mechanisms;<br>General function prediction only;<br>Energy production and conversion;<br>Amino acid transport and metabolism;<br>Carbohydrate transport and metabolism;<br>Signal transduction mechanisms;<br>Coenzyme transport and metabolism;<br>Secondary metabolites biosynthesis, transport and catabolism;<br>Inorganic ion transport and metabolism;<br>Posttranslational modification, protein turnover, chaperones;<br>Cell wall/membrane/envelope biogenesis;<br>Cell motility;<br>Lipid transport and metabolism;<br>Extracellular structures;<br>RNA processing and modification;<br>Cytoskeleton;<br>Nuclear structure
**KEGG**	Genetic Information Processing;<br>Metabolism;<br>Nucleotide Metabolism;<br>Digestive System;<br>Enzyme Families;<br>Poorly Characterized;<br>Amino Acid Metabolism;<br>Biosynthesis of Other Secondary Metabolites;<br>Cell Growth and Death;<br>Folding, Sorting and Degradation;<br>Metabolism of Cofactors and Vitamins;<br>Circulatory System;<br>Carbohydrate Metabolism;<br>Energy Metabolism;<br>Transcription;<br>Membrane Transport;<br>Glycan Biosynthesis and Metabolism;<br>Translation;<br>Lipid Metabolism;<br>Metabolism of Other Amino Acids;<br>Xenobiotics Biodegradation and Metabolism;<br>Cellular Processes and Signaling;<br>Sensory System;<br>Replication and Repair;<br>Signal Transduction;<br>Cell Motility;<br>Metabolism of Terpenoids and Polyketides;<br>Transport and Catabolism;<br>Infectious Diseases;<br>Endocrine System;<br>Signaling Molecules and Interaction;<br>Excretory System;<br>Environmental Adaptation;<br>Cancers;<br>Immune System Diseases;<br>Nervous System;<br>Immune System

Table S15A. The number of various *trios* in the class "*Trios without MAO handle*" in the MF/MP (Metagenome Functions/Pathways) networks with FDR control

Disease & Treatments		Trios with MAO 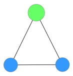									Trios without MAO 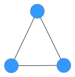				
		+ – – Type-1		Σ	+ + – Type-2		Σ	Type-3 – – –	Type-4 + + +	Σ	– – –	+ – –	+ + –	+ + +	Σ
		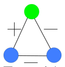 Type-1A	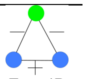 Type-1B		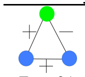 Type-2A	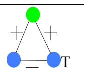 Type-2B									
**MP Type-I (KEGG)**															
Stool	Stool	15	15	30	0	3	3	1	7	41	5	116	29	72	222
Obesity	Lean	19	22	41	1	3	4	2	3	50	5	121	16	67	209
	Overweight	20	7	27	0	1	1	0	5	33	5	133	27	68	233
Type 2 diabetes	Healthy	19	12	31	1	2	3	0	7	41	2	74	17	53	146
	Disease	6	1	7	1	2	3	0	15	25	3	77	19	16	115
IBD	Healthy	12	12	24	2	0	2	0	3	29	8	133	13	79	233
	Disease	7	17	24	1	2	3	0	0	27	10	103	20	59	192
**MP Type-II (KEGG)**															
Stool	Stool	32	18	50	0	0	0	0	7	57	5	184	12	120	321
Obesity	Lean	15	11	26	0	0	0	0	3	29	2	164	14	105	285
	Overweight	11	13	24	0	2	2	2	1	29	13	166	21	44	244
Type 2 diabetes	Healthy	10	5	15	0	3	3	2	3	23	0	105	15	48	168
	Disease	2	3	5	0	0	0	1	1	7	1	73	20	37	131
IBD	Healthy	2	1	3	0	0	0	0	0	3	1	75	13	52	141
	Disease	3	5	8	0	0	0	1	0	9	6	106	14	34	160
**MF Type-I (eggNOG)**															
Stool	Stool	3	6	9	0	2	2	3	0	14	3	35	10	9	57
Obesity	Lean	7	2	9	0	0	0	2	2	13	9	47	5	8	69
	Overweight	8	0	8	0	1	1	1	7	17	4	36	10	6	56
Type 2 diabetes	Healthy	2	6	8	1	2	3	5	1	17	0	19	5	7	31
	Disease	2	5	7	0	1	1	1	0	9	0	27	6	4	37
IBD	Healthy	5	0	5	0	0	0	0	1	6	3	39	4	7	53
	Disease	7	2	9	0	0	0	3	4	16	2	30	13	11	56
**MF Type-II (eggNOG)**															
Stool	Stool	7	4	11	0	1	1	1	0	13	3	89	16	39	147
Obesity	Lean	3	0	3	2	1	3	0	1	7	2	24	6	12	44
	Overweight	1	3	4	0	0	0	1	0	5	1	36	14	10	61
Type 2 diabetes	Healthy	2	2	4	0	0	0	0	0	4	1	33	10	5	49
	Disease	0	6	6	0	0	0	0	0	6	4	25	11	11	51
IBD	Healthy	0	0	0	0	0	0	0	0	0	1	38	7	9	55
	Disease	4	5	9	0	1	1	0	0	10	1	32	7	14	54

Table S15B. Randomization test for the number of various *trios* in the class "*Trios without MAO handle*" in the MF/MP (Metagenome Functions/Pathways) networks with FDR control

Microbiomes & Associated Disease Treatments		Trios with MAO									Trios without MAO				
		Type-1 (+ – –)			Type-2 (+ + –)			Type-3	Type-4						
								–	+		–	+	+	+	
								–	+		–	–	+	+	
		Type-1A	Type-1B	Σ	Type-2A	Type-2B	Σ	–	+	Σ	–	–	–	+	Σ
**MP Type-I (KEGG)**															
Obesity	Lean vs. Overweight	0.98	0.58	0.70	0.68	0.63	0.48	0.37	0.73	0.70	1.00	0.94	0.54	1.00	0.92
Type 2 diabetes	Healthy vs. Disease	0.59	0.25	0.37	1.00	1.00	1.00	1.00	0.65	0.71	0.90	1.00	0.83	0.39	0.85
IBD	Healthy vs. Disease	0.76	0.89	1.00	0.26	0.24	0.69	1.00	0.56	1.00	0.51	0.86	0.30	0.81	0.87
**MP Type-II (KEGG)**															
Obesity	Lean vs. Overweight	0.84	0.95	0.92	1.00	0.27	0.27	0.61	0.87	1.00	0.05	1.00	0.58	0.57	0.89
Type 2 diabetes	Healthy vs. Disease	0.72	0.92	0.83	1.00	0.00	0.00	0.53	0.82	0.73	0.79	0.78	0.36	0.81	0.83
IBD	Healthy vs. Disease	0.99	0.87	0.92	1.00	1.00	1.00	0.05	1.00	0.86	0.24	0.75	0.94	0.79	0.89
**MF Type-I (eggNOG)**															
Obesity	Lean vs. Overweight	0.98	0.88	0.99	1.00	0.79	0.84	0.82	0.78	0.89	0.42	0.92	0.51	0.96	0.91
Type 2 diabetes	Healthy vs. Disease	1.00	0.90	0.96	0.59	0.56	0.36	0.14	0.75	0.66	1.00	0.89	0.90	0.85	0.93
IBD	Healthy vs. Disease	0.89	0.84	0.82	1.00	1.00	1.00	0.00	0.75	0.67	0.85	0.94	0.08	0.91	1.00
**MF Type-II (eggNOG)**															
Obesity	Lean vs. Overweight	0.83	0.66	0.99	0.10	0.87	0.23	0.49	0.71	0.96	0.87	0.82	0.28	0.95	0.85
Type 2 diabetes	Healthy vs. Disease	0.63	0.68	0.92	1.00	1.00	1.00	1.00	1.00	0.93	0.30	0.89	0.93	0.62	0.99
IBD	Healthy vs. Disease	0.36	0.55	0.40	1.00	0.17	0.19	1.00	1.00	0.38	1.00	0.81	1.00	0.81	1.00

Table S15C. The number of “Trios with MAO handle” in the MF/MP (Metagenome Functions/Pathways) networks with FDR control

Disease & Treatments		Single-Link MAO 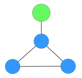			Double-Link MAO 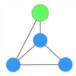				Triple-Link MAO 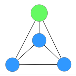				
		–	+	∑	– –	+ –	+ +	∑	– – –	+ – –	+ + –	+ + +	∑
**MP Type-I (KEGG)**													
Stool	Stool	66	27	93	31	31	12	74	6	11	6	2	25
Obesity	Lean	35	12	47	39	31	4	74	12	25	8	0	45
	Overweight	74	24	98	20	42	5	67	0	9	10	0	19
Type 2 diabetes	Healthy	19	17	36	24	20	8	52	8	18	10	0	36
	Disease	12	35	47	0	15	31	46	0	1	1	3	5
IBD	Healthy	73	27	100	46	23	10	79	9	4	5	0	18
	Disease	67	15	82	34	26	4	64	14	8	2	0	24
**MP Type-II (KEGG)**													
Stool	Stool	55	16	71	43	47	8	98	16	60	42	4	122
Obesity	Lean	85	30	115	39	40	10	89	6	11	6	0	23
	Overweight	91	24	115	40	31	2	73	3	4	1	0	8
Type 2 diabetes	Healthy	56	11	67	8	15	3	26	0	0	5	0	5
	Disease	47	20	67	3	5	4	12	0	0	0	0	0
IBD	Healthy	49	19	68	2	4	0	6	0	0	0	0	0
	Disease	64	17	81	17	6	0	23	0	0	0	0	0
**MF Type-I (eggNOG)**													
Stool	Stool	16	8	24	15	10	0	25	1	4	0	0	5
Obesity	Lean	9	18	27	2	9	7	18	0	2	2	0	4
	Overweight	9	14	23	3	13	5	21	0	1	6	1	8
Type 2 diabetes	Healthy	13	0	13	11	1	0	12	3	0	1	0	4
	Disease	16	6	22	2	6	0	8	0	0	0	0	0
IBD	Healthy	10	9	19	0	7	1	8	0	0	1	0	1
	Disease	14	13	27	10	9	3	22	0	0	1	1	2
**MF Type-II (eggNOG)**													
Stool	Stool	33	14	47	10	18	0	28	1	6	0	0	7
Obesity	Lean	4	15	19	0	4	8	12	0	0	1	0	1
	Overweight	11	20	31	7	4	0	11	0	0	0	0	0
Type 2 diabetes	Healthy	14	7	21	2	5	0	7	0	1	0	0	1
	Disease	16	0	16	8	0	0	8	2	0	0	0	2
IBD	Healthy	2	13	15	0	0	0	0	0	0	0	0	0
	Disease	13	7	20	6	9	0	15	2	3	0	0	5

Table S15D. Randomization test for the number of “Trios with MAO handle” in the MF/MP (Metagenome Functions/Pathways) networks with FDR control

Microbiomes & Associated Disease Treatments		Single-Link MAO 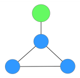			Double-Link MAO 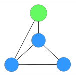				Triple-Link MAO 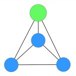				
		–	+	∑	– –	+ –	+ +	∑	– – –	+ – –	+ + –	+ + +	∑
**MP Type-I (KEGG)**													
Obesity	Lean vs. Overweight	0.30	0.84	0.55	0.86	0.86	0.91	0.98	0.92	0.84	0.99	1.00	0.92
Type 2 diabetes	Healthy vs. Disease	0.75	0.43	0.82	0.45	0.92	0.11	0.98	0.73	0.84	0.93	0.91	0.88
IBD	Healthy vs. Disease	0.87	0.49	0.67	0.79	0.96	0.51	0.85	0.94	0.95	0.93	1.00	0.99
**MP Type-II (KEGG)**													
Obesity	Lean vs. Overweight	0.89	0.72	1.00	0.95	0.85	0.56	0.87	0.99	0.98	0.94	1.00	0.93
Type 2 diabetes	Healthy vs. Disease	0.74	0.45	1.00	0.95	0.85	0.97	0.92	1.00	1.00	0.95	1.00	0.98
IBD	Healthy vs. Disease	0.57	0.90	0.66	0.55	0.97	1.00	0.83	1.00	1.00	1.00	1.00	1.00
**MF Type-I (eggNOG)**													
Obesity	Lean vs. Overweight	1.00	0.96	0.95	0.99	0.88	0.97	0.97	1.00	0.98	0.96	0.98	0.97
Type 2 diabetes	Healthy vs. Disease	0.98	0.68	0.88	0.77	0.95	1.00	0.96	0.81	1.00	0.98	1.00	0.89
IBD	Healthy vs. Disease	0.93	0.96	0.93	0.68	0.96	0.93	0.87	1.00	1.00	1.00	0.90	1.00
**MF Type-II (eggNOG)**													
Obesity	Lean vs. Overweight	0.82	0.91	0.86	0.71	1.00	0.69	1.00	1.00	1.00	0.93	1.00	0.96
Type 2 diabetes	Healthy vs. Disease	0.96	0.83	0.92	0.82	0.79	1.00	1.00	0.85	0.97	1.00	1.00	1.00
IBD	Healthy vs. Disease	0.67	0.65	0.91	0.70	0.40	1.00	0.49	0.80	0.60	1.00	1.00	0.64

Table S16A. The P/N (positive to negative links) ratios in the MF/MP (Metagenome Functions/Pathways) networks with FDR control

Disease	Treatments	Positive Links (+)	Negative Links (–)	P/N (+/–) Ratio
**MP Type-I (KEGG)**				
Stool	Stool	112	90	1.244
Obesity	Lean	106	96	1.104
	Overweight	106	95	1.116
Type 2 diabetes	Healthy	105	69	1.522
	Disease	82	82	1.000
IBD	Healthy	105	90	1.167
	Disease	98	84	1.167
**MP Type-II (KEGG)**				
Stool	Stool	117	90	1.300
Obesity	Lean	117	87	1.345
	Overweight	96	110	0.873
Type 2 diabetes	Healthy	90	85	1.059
	Disease	87	68	1.279
IBD	Healthy	91	78	1.167
	Disease	86	87	0.989
**MF Type-I (eggNOG)**				
Stool	Stool	40	49	0.816
Obesity	Lean	35	58	0.603
	Overweight	44	47	0.936
Type 2 diabetes	Healthy	31	41	0.756
	Disease	34	45	0.756
IBD	Healthy	34	52	0.654
	Disease	46	45	1.022
**MF Type-II (eggNOG)**				
Stool	Stool	57	53	1.075
Obesity	Lean	39	37	1.054
	Overweight	42	39	1.077
Type 2 diabetes	Healthy	35	42	0.833
	Disease	38	37	1.027
IBD	Healthy	41	39	1.051
	Disease	44	41	1.073

Table S16B. Randomization test for the P/N (positive to negative links) ratios in the MF/MP (Metagenome Functions/Pathways) networks with FDR control

**Microbiome**	**Treatments**	**Positive Links (+)**	**Negative Links (–)**	**P/N (+/–) Ratio**
		**MP Type-I (KEGG)**		
Obesity	Lean vs. Overweight	1.00	0.96	0.90
Type 2 diabetes	Healthy vs. Disease	0.13	0.28	0.00
IBD	Healthy vs. Disease	0.68	0.62	1.00
		**MP Type-II (KEGG)**		
Obesity	Lean vs. Overweight	0.22	0.23	0.00
Type 2 diabetes	Healthy vs. Disease	0.86	0.07	0.01
IBD	Healthy vs. Disease	0.79	0.51	0.09
		**MF Type-I (eggNOG)**		
Obesity	Lean vs. Overweight	0.25	0.23	0.00
Type 2 diabetes	Healthy vs. Disease	0.55	0.61	1.00
IBD	Healthy vs. Disease	0.22	0.63	0.00
		**MF Type-II (eggNOG)**		
Obesity	Lean vs. Overweight	0.64	0.88	0.77
Type 2 diabetes	Healthy vs. Disease	0.72	0.59	0.06
IBD	Healthy vs. Disease	0.73	0.84	0.80

Table S17 (MS-Excel Table). The list of Core/Periphery nodes from the MF/MP (metagenome function / metagenome pathway) networks of the human gut metagenomes

Table S18A. The core/periphery and nested structures in the MF/MP (Metagenome Functions/Pathways) networks with FDR control

Disease	*Treatments*	ρ	Ratio of C/(C+P)	Density Matrix			Nestedness (S)
				B11	B12 (21)	B22	
		**MP Type-I (KEGG)**					
Stool	Stool	0.498	0.139	0.400	0.948	1.000	0.998
Obesity	Lean	0.705	0.111	0.500	1.000	1.000	1.000
	Overweight	0.420	0.222	0.607	0.951	1.000	0.996
Type 2 diabetes	Healthy	0.694	0.972	0.953	0.000	0.000	0.974
	Disease	0.816	0.091	0.333	1.000	1.000	1.000
IBD	Healthy	0.573	0.167	0.667	1.000	1.000	1.000
	Disease	0.704	0.972	0.971	0.000	0.000	0.990
		**MP Type-II (KEGG)**					
Stool	Stool	0.683	0.806	0.941	0.276	0.476	0.864
Obesity	Lean	0.777	0.889	0.984	0.242	1.000	0.932
	Overweight	0.917	0.861	0.987	0.052	0.600	0.858
Type 2 diabetes	Healthy	0.840	0.829	0.961	0.115	0.333	0.770
	Disease	0.795	0.879	0.958	0.181	0.000	0.957
IBD	Healthy	0.863	0.889	0.960	0.031	1.000	0.842
	Disease	0.770	0.917	0.941	0.111	0.333	0.940
		**MF Type-I (eggNOG)**					
Stool	Stool	0.625	0.208	0.600	1.000	1.000	0.999
Obesity	Lean	0.416	0.417	0.800	1.000	1.000	0.999
	Overweight	1.000	0.083	0.000	1.000	1.000	1.000
Type 2 diabetes	Healthy	0.933	0.917	1.000	0.114	0.000	0.996
	Disease	0.949	0.957	1.000	0.000	0.000	0.998
IBD	Healthy	0.952	0.958	1.000	0.000	0.000	0.998
	Disease	0.000	1.000	1.000	0.000	0.000	1.000
		**MF Type-II (eggNOG)**					
Stool	Stool	0.562	0.833	0.979	0.525	0.667	0.941
Obesity	Lean	0.850	0.958	1.000	0.000	0.000	0.998
	Overweight	0.905	0.958	0.992	0.000	0.000	0.993
Type 2 diabetes	Healthy	0.583	0.870	0.995	0.583	0.333	0.968
	Disease	0.681	0.913	0.957	0.310	0.000	0.970
IBD	Healthy	0.779	0.958	0.964	0.000	0.000	0.984
	Disease	0.605	0.167	0.167	0.925	1.000	0.997

Table S18B. Randomization test for the core/periphery and nested structures in the MF/MP (Metagenome Functions/Pathways) networks with FDR control

Disease	***Treatments***	***ρ***	**Ratio of C/(C+P)**	**Density Matrix**			**Nestedness (*S*)**
				B11	B12(21)	B22	
			**MP Type-I (KEGG)**				
Obesity	Lean vs. Overweight	0.00	0.50	0.44	0.55	1.00	0.94
Type 2 diabetes	Healthy vs. Disease	0.12	0.00	0.00	0.00	0.00	0.80
IBD	Healthy vs. Disease	0.18	0.00	0.08	0.00	0.00	0.87
			**MP Type-II (KEGG)**				
Obesity	Lean vs. Overweight	0.06	0.76	0.99	0.06	0.05	0.19
Type 2 diabetes	Healthy vs. Disease	0.59	0.37	0.97	0.24	0.00	0.01
IBD	Healthy vs. Disease	0.21	0.74	0.95	0.22	0.00	0.12
			**MF Type-I (eggNOG)**				
Obesity	Lean vs. Overweight	0.00	0.00	0.00	1.00	1.00	0.99
Type 2 diabetes	Healthy vs. Disease	0.81	0.79	1.00	0.02	1.00	0.97
IBD	Healthy vs. Disease	0.00	0.52	1.00	1.00	1.00	0.97
			**MF Type-II (eggNOG)**				
Obesity	Lean vs. Overweight	0.62	1.00	0.97	1.00	1.00	0.92
Type 2 diabetes	Healthy vs. Disease	0.12	0.74	0.67	0.00	0.02	0.96
IBD	Healthy vs. Disease	0.09	0.00	0.00	0.00	0.00	0.88

Table S19. The shared MP/MF analysis between the healthy and diseased treatments

Microbiome	Treatments	Reads randomization (A1)*		Samples randomization (A2)*	
		Core**	Total	Core	Total
		**MP Type-I (KEGG)**			
Obesity	Lean vs. Overweight	1.000	1.000	1.000	1.000
Type 2 diabetes	Healthy vs. Disease	1.000	0.554	1.000	0.698
IBD	Healthy vs. Disease	1.000	1.000	1.000	1.000
		**MP Type-II (KEGG)**			
Obesity	Lean vs. Overweight	1.000	1.000	1.000	1.000
Type 2 diabetes	Healthy vs. Disease	1.000	1.000	1.000	0.632
IBD	Healthy vs. Disease	1.000	1.000	1.000	1.000
		**MF Type-I (eggNOG)**			
Obesity	Lean vs. Overweight	1.000	1.000	1.000	1.000
Type 2 diabetes	Healthy vs. Disease	1.000	0.129	1.000	0.501
IBD	Healthy vs. Disease	1.000	1.000	1.000	1.000
		**MF Type-II (eggNOG)**			
Obesity	Lean vs. Overweight	1.000	1.000	1.000	1.000
Type 2 diabetes	Healthy vs. Disease	1.000	1.000	1.000	0.524
IBD	Healthy vs. Disease	1.000	1.000	1.000	1.000

*Algorithm **A1** is randomization reassignments (remix) of all reads across samples and MFGCs Algorithm **A2** is randomization reassignments (remix) of all samples only. *See* Ma *et al*. (2019) ISME Journal or Ma (2020) for detailed introduction of **A1** & **A2**.

*Since there were only two periphery nodes, it was not possible to perform shared periphery analysis.

Table S20A. Statistical properties of the high-salience skeletons in the MF/MP (Metagenome Functions/Pathways) networks with FDR control

**Disease**	**Treatments**	**Statistics of HSS**						Assortativity
		Links (%)	Max	Mean	Std. Err	Skewness	Kurtosis	r_{HSS}
**MP Type-I (KEGG)**								
Stool	Stool	81.270	0.917	0.056	0.074	8.897	96.362	-0.029
Obesity	Lean	97.619	0.278	0.056	0.014	5.721	98.810	-0.029
	Overweight	87.778	0.861	0.056	0.053	11.448	170.038	-0.029
Type 2 diabetes	Healthy	89.524	0.806	0.056	0.041	10.568	180.588	-0.029
	Disease	99.683	0.111	0.056	0.004	-2.882	158.983	-0.029
IBD	Healthy	89.841	0.861	0.056	0.050	12.595	198.274	-0.029
	Disease	90.991	0.730	0.054	0.036	10.700	189.835	-0.028
**MP Type-II (KEGG)**								
Stool	Stool	61.429	0.694	0.056	0.076	3.968	23.966	-0.029
Obesity	Lean	78.254	0.833	0.056	0.074	7.758	74.552	-0.029
	Overweight	73.810	1.000	0.056	0.078	7.362	73.221	-0.029
Type 2 diabetes	Healthy	86.667	0.583	0.056	0.043	7.133	80.122	-0.029
	Disease	76.984	0.861	0.056	0.062	6.574	65.997	-0.029
IBD	Healthy	75.079	0.972	0.056	0.079	8.349	87.445	-0.029
	Disease	79.580	0.919	0.054	0.063	9.118	112.198	-0.028
**MF Type-I (eggNOG)**								
Stool	Stool	92.754	0.875	0.083	0.054	11.304	169.025	-0.043
Obesity	Lean	96.377	0.500	0.083	0.031	8.456	124.009	-0.043
	Overweight	92.754	0.875	0.083	0.054	11.304	169.025	-0.043
Type 2 diabetes	Healthy	85.145	1.000	0.083	0.080	8.334	89.169	-0.043
	Disease	92.095	0.957	0.087	0.061	11.249	162.481	-0.045
IBD	Healthy	86.594	0.958	0.083	0.077	8.043	84.547	-0.043
	Disease	99.638	0.125	0.083	0.006	-6.819	137.500	-0.043
**MF Type-II (eggNOG)**								
Stool	Stool	73.913	0.667	0.083	0.075	2.540	14.570	-0.043
Obesity	Lean	92.391	0.917	0.083	0.057	11.526	173.002	-0.043
	Overweight	90.942	0.625	0.083	0.048	6.083	66.447	-0.043
Type 2 diabetes	Healthy	82.971	0.750	0.083	0.068	4.859	40.821	-0.043
	Disease	79.842	0.739	0.087	0.078	4.671	35.048	-0.045
IBD	Healthy	86.594	0.917	0.083	0.068	7.660	88.662	-0.043
	Disease	92.754	0.292	0.083	0.031	0.514	10.410	-0.043

Table S20B. Randomization test for statistical properties of the high-salience skeletons in the MF/MP (Metagenome Functions/Pathways) networks with FDR control

**Microbiome**	**Treatments**	**Statistics of HSS**						Assortativity
		Links (%)	Max	Mean	Std. Err	Skewness	Kurtosis	r_{HSS}
**MP Type-I (KEGG)**								
Obesity	Lean vs. Overweight	0.00	0.00	1.00	0.00	0.00	0.00	1.00
Type 2 diabetes	Healthy vs. Disease	0.48	0.00	1.00	0.01	0.00	0.00	1.00
IBD	Healthy vs. Disease	0.82	0.44	0.13	0.15	0.01	0.32	0.13
**MP Type-II (KEGG)**								
Obesity	Lean vs. Overweight	0.43	0.45	1.00	0.68	0.66	0.92	1.00
Type 2 diabetes	Healthy vs. Disease	0.54	0.07	1.00	0.46	0.43	0.04	1.00
IBD	Healthy vs. Disease	0.28	0.55	0.10	0.19	0.28	0.00	0.10
**MF Type-I (eggNOG)**								
Obesity	Lean vs. Overweight	0.53	0.08	1.00	0.03	0.00	0.00	1.00
Type 2 diabetes	Healthy vs. Disease	0.05	0.92	0.00	0.04	0.00	0.00	0.02
IBD	Healthy vs. Disease	0.01	0.00	1.00	0.00	0.00	0.00	1.00
**MF Type-II (eggNOG)**								
Obesity	Lean vs. Overweight	0.84	0.08	1.00	0.53	0.00	0.00	1.00
Type 2 diabetes	Healthy vs. Disease	0.59	0.79	0.03	0.49	0.83	0.41	0.67
IBD	Healthy vs. Disease	0.26	0.00	1.00	0.01	0.00	0.00	1.00

Table S21. The similarity (*C*) and normalized stochasticity ratio (NSR) for the intra-healthy, intra-diseased, and inter-healthy-and-diseased treatments for the human gut metagenome

**Metagenomic Operational Unit**	**Treatments of Disease Cases**	**Similarity (*C*)**			**NSR**		
		**Intra-H**	**Intra-D**	**Inter-H&D**	**Intra-H**	**Intra-D**	**Inter-H&D**
MG	Obesity	0.747	0.710	0.732	0.213	0.196	0.204
	Type-2 Diabetes	0.657	0.676	0.666	0.179	0.169	0.170
	IBD	0.686	0.693	0.691	0.205	0.187	0.189
Type-I MFGC based on KEGG	Obesity	0.965	0.967	0.964	0.933	0.936	0.932
	Type-2 Diabetes	0.958	0.970	0.971	0.919	0.943	0.945
	IBD	0.971	0.962	0.969	0.938	0.930	0.939
Type-II MFGC based on KEGG	Obesity	0.924	0.911	0.925	0.872	0.863	0.877
	Type-2 Diabetes	0.824	0.802	0.812	0.772	0.751	0.760
	IBD	0.867	0.871	0.884	0.803	0.815	0.831
Type-I MFGC based on eggNOG	Obesity	0.955	0.957	0.955	0.935	0.940	0.936
	Type-2 Diabetes	0.946	0.960	0.961	0.923	0.937	0.941
	IBD	0.963	0.951	0.958	0.947	0.932	0.942
Type-II MFGC based on eggNOG	Obesity	0.906	0.890	0.907	0.841	0.832	0.848
	Type-2 Diabetes	0.789	0.766	0.774	0.726	0.705	0.712
	IBD	0.846	0.842	0.856	0.773	0.773	0.791
Mean	Obesity	0.899	0.887	0.897	0.759	0.753	0.759
	Type-2 Diabetes	0.835	0.835	0.837	0.704	0.701	0.706
	IBD	0.867	0.864	0.872	0.733	0.727	0.738

Table S22. The *p*-value of Wilcoxon test of the normalized stochasticity ratio (NSR) for the pair-wise comparison of the intra-healthy treatment, intra-diseased treatment and inter-healthy & diseased treatments of the human gut metagenomes

Metagenomic Unit	Treatments of Disease Cases	Intra-H vs. Intra-D			Intra-H vs. Inter H & D			Intra-D vs. Inter H & D		
		≠	>	<	≠	>	<	≠	>	<
MG	Obesity	0.000	0.000	1.000	0.020	0.010	0.990	0.016	0.992	0.008
	Type-2 Diabetes	0.112	0.056	0.944	0.100	0.050	0.950	0.794	0.603	0.397
	IBD	0.019	0.009	0.991	0.023	0.012	0.988	0.603	0.699	0.301
Type-I MFGC based on KEGG	Obesity	0.000	1.000	0.000	0.000	1.000	0.000	0.000	1.000	0.000
	Type-2 Diabetes	0.000	0.000	1.000	0.000	0.000	1.000	0.000	0.000	1.000
	IBD	0.157	0.921	0.079	0.000	1.000	0.000	0.000	1.000	0.000
Type-II MFGC based on KEGG	Obesity	0.000	0.000	1.000	0.006	0.003	0.997	0.000	1.000	0.000
	Type-2 Diabetes	0.000	0.000	1.000	0.045	0.977	0.023	0.029	0.015	0.985
	IBD	0.000	0.000	1.000	0.042	0.021	0.979	0.008	0.996	0.004
Type-I MFGC based on eggNOG	Obesity	0.000	0.000	1.000	0.000	1.000	0.000	0.000	1.000	0.000
	Type-2 Diabetes	0.128	0.936	0.064	0.010	0.005	0.995	0.585	0.292	0.708
	IBD	0.201	0.100	0.900	0.005	0.997	0.003	0.000	1.000	0.000
Type-II MFGC based on eggNOG	Obesity	0.000	0.000	1.000	0.000	0.000	1.000	0.000	1.000	0.000
	Type-2 Diabetes	0.000	0.000	1.000	0.082	0.959	0.041	0.007	0.003	0.997
	IBD	0.000	0.000	1.000	0.045	0.022	0.978	0.012	0.994	0.006
% With Significant Differences	Obesity	100% (5/5)	80% (4/5)	20% (1/5)	100% (5/5)	60% (3/5)	40% (2/5)	100% (5/5)	0% (0/5)	100% (5/5)
	Type-2 Diabetes	60% (3/5)	60% (3/5)	0% (0/5)	60% (3/5)	40% (2/5)	40% (2/5)	60% (3/5)	60% (3/5)	0% (0/5)
	IBD	60% (3/5)	60% (3/5)	0% (0/5)	100% (5/5)	60% (3/5)	40% (2/5)	80% (4/5)	0% (0/5)	80% (4/5)
